# Measuring the shadows: A systematic review of chronic emptiness in borderline personality disorder

**DOI:** 10.1371/journal.pone.0233970

**Published:** 2020-07-01

**Authors:** Caitlin E. Miller, Michelle L. Townsend, Nicholas J. S. Day, Brin F. S. Grenyer

**Affiliations:** 1 School of Psychology, University of Wollongong, Wollongong, NSW, Australia; 2 Illawarra Health and Medical Research Institute, University of Wollongong, Wollongong, NSW, Australia; Medical University of Vienna, AUSTRIA

## Abstract

**Background:**

Chronic feelings of emptiness is an under-researched symptom of borderline personality disorder (BPD), despite indications it may be central to the conceptualisation, course, and outcome of BPD treatment. This systematic review aimed to provide a comprehensive overview of chronic feelings of emptiness in BPD, identify key findings, and clarify differences between chronic feelings of emptiness and related constructs like depression, hopelessness, and loneliness.

**Method:**

A PRISMA guided systematic search of the literature identified empirical studies with a focus on BPD or BPD symptoms that discussed chronic feelings of emptiness or a related construct.

**Results:**

Ninety-nine studies met criteria for inclusion in the review. Key findings identified there were significant difficulties in defining and measuring chronic emptiness. However, based on the studies reviewed, chronic emptiness is a sense of disconnection from both self and others. When experienced at frequent and severe levels, it is associated with low remission for people with BPD. Emptiness as a construct can be separated from hopelessness, loneliness and intolerance of aloneness, however more research is needed to explicitly investigate these experiences. Chronic emptiness may be related to depressive experiences unique to people with BPD, and was associated with self-harm, suicidality, and lower social and vocational function.

**Conclusions and implications:**

We conclude that understanding chronic feelings of emptiness is central to the experience of people with BPD and treatment focusing on connecting with self and others may help alleviate a sense of emptiness. Further research is required to provide a better understanding of the nature of chronic emptiness in BPD in order to develop ways to quantify the experience and target treatment.

Systematic review registration number: CRD42018075602.

## Introduction

‘To define accurately what the word [emptiness] means in any context can feel like trying to find a light switch in a totally dark and unfamiliar room’ [1, p. 331].

Borderline personality disorder (BPD) is a complex mental disorder characterised by a pervasive instability of self-concept, emotions, and behaviour [2]. Globally, lifetime prevalence of BPD is estimated at approximately 6% [3], but individuals with BPD can account for up to 20.5% of emergency department presentations and 26.6% of inpatient psychological services [4]. Within personality disorder research, the landscape of formulation and diagnosis is evolving, and there is a need to research features of BPD which are important in both traditional categorical and emerging dimensional approaches [5]. Current diagnosis for BPD involves identifying a minimum five of nine possible criteria in the Diagnostic and Statistical Manual of Mental Disorders (DSM-5) [2]. One criterion is labelled chronic feelings of emptiness. This symptom remains in the alternative diagnostic model for BPD in DSM-5, where it is associated with identity disturbance.

Feelings of chronic emptiness have always been included in the conceptualisation and diagnosis of BPD [6]. In an early seminal paper, Deutsch [7] described a group of people who experience inner emptiness in their emotional life, a feeling where ‘all inner experience is completely excluded. It is like the performance of an actor who is technically well trained but who lacks the necessary spark to make his impersonations true to life’ [7, p. 328]. This experience was described as resulting in a ‘chameleonlike quality’ in interpersonal relationships, where pretence and adaptability masks the emptiness underneath [8]. Chronic feelings of emptiness has also been described as akin to ‘deadness’, ‘nothingness’, a ‘void’, feeling ‘swallowed’[9], a sense of ‘vagueness’ [10], a feeling of internal absence [11], ‘woodenness’ [12], a ‘hole’ or ‘vacuum’, ‘aloneness’ [1], ‘isolation’ [13], ‘numbness’ and ‘alienation’[14].

There are several theoretical views of chronic emptiness in BPD. According to early theoretical literature, people who experience chronic feelings of emptiness lack the capacity to experience themselves, others, or the world fully and there is ‘a profound lack of emotional depth or sense of not being in the experience’ [9, p. 34, 11]. Kernberg [8, 15] suggested that emptiness results from a loss of, or disturbance in, the relationship of self with object relations, with a lack of integrated representations leading to an absence of ‘self-feeling’ [16, 17]. Other analysts similarly proposed that emptiness results from deficits in maintaining stable object relations [18–20] and an inability to develop soothing and holding introjects–meaning difficulties with internalising positive and nurturing experiences [21, 22], perhaps resulting from the absence of a ‘good enough’ caregiver [23, 24]. Overall, these analysts attributed emptiness to the absence of a good maternal presence, resulting in unstable object- and self-representations and a feeling of inner emptiness. This theory was supported in part by an early study by Grinker and colleagues [25] which found inadequate awareness of self was sufficient for predicting BPD group membership, including a deficiency in recognising internal thoughts and affects as belonging to oneself and an associated feeling of chronic emptiness. Chronic feelings of emptiness were proposed to drive ‘the basis of his attempt to appropriate from others, or of his feeling of danger of being engulfed by others. Some try to borrow from others, become satellitic to another, merge with a host or lay skin to skin. Others attempt to fill up with knowledge or experience’ [25, p. 16]. These early concepts are still utilised within contemporary psychodynamic approaches to personality assessment, diagnosis and treatment, with a focus on emptiness reflecting disturbances of identity [26, 27].

Biosocial models of BPD suggest that chronic feelings of emptiness are reflective of a dysregulation of identity [28]. Emptiness is conceptualised as an attempt (whether conscious or not) to inhibit intense emotional experiences, which leads to a lack of development in personal identity [29]. It is hypothesised chronic emptiness results from insecure attachments with caregivers [30], and transactional models propose emptiness is the experience of an individual not knowing their own personal experience, resulting from inconsistent validation and invalidation responses by caregivers [31]. This understanding is similar to attachment and mentalisation perspectives, where feelings of emptiness reflect a failure in mentalisation. Specifically, emptiness is a consequence of the absence of the psychological self–the secondary representation of self which allows an understanding of one’s own internal world, and the world seen through the eyes of others [32].

Despite the numerous theories that mention emptiness, there remains no unifying theory of chronic emptiness in BPD, and it is not typically accounted for in aetiological models of BPD [33]. Further, the symptom has rarely been the focus of empirical research [1]. Substantial empirical literature exists for other symptoms of BPD (e.g. affective instability [34] and impulsivity [35]), but until recently there has been a limited focus on chronic feelings of emptiness. There appears to be confusion within the field regarding what chronic emptiness actually *is*, with vague boundaries between constructs like hopelessness, loneliness, or boredom [36] and with research often referring to each term interchangeably.

Despite this lack of clarity within the research, recent studies have shown an increased focus on chronic emptiness, suggesting the experience may be associated with vocational and interpersonal dysfunction [37, 38] and self-harm and suicidal behaviours [39]. Research has also linked chronic emptiness to depressive experiences unique to people with BPD–a possible ‘borderline depression’ [40].

In order to better understand what chronic emptiness is and the importance of chronic feelings of emptiness to the conceptualisation, course, and outcomes of BPD it is important to first analyse the current literature to provide a baseline for future work. In particular, it is important to identify any research that supports theoretical claims that chronic emptiness is a reflection of impaired relationships with the self and others. It is also important to identify research that reports on whether chronic emptiness represents a single construct or if it encompasses other experiences, such hopelessness, loneliness or depression. In order to achieve this, the current study aimed to systematically review empirical research on chronic emptiness and related terms in populations with features or a diagnosis of BPD. Considering there are currently no detailed reviews, a broad focus was employed that is unrestrictive to interventions and outcomes. A cohesive analysis of the empirical literature will enable an understanding of the current state of the field, and provide directions for future research.

## Method

### Protocol and registration

A protocol for the current study was registered on the International Prospective Register of Systematic Reviews (PROSPERO, registration number: CRD42018075602). Articles were identified, screened, and chosen for inclusion in accordance with the Preferred Reporting Items for Systematic Review and Meta-Analysis (PRISMA) guidelines for reviews [41].

### Information sources

Electronic databases searched included PsycINFO, PubMed, Scopus, and Web of Science. The last search date was February 2019. Additional records known to authors which were not captured in original database searching were added.

### Search

The search strategy for online studies remained the same across databases and included (Empt* or isolat* or vacuum or dead or deadness or nothing* or void or swallowed or bored* or numb* or alien* or wooden* or hole or alone* or vague* or hopeless* or lonel*) AND (borderline personality disorder or BPD or emotionally unstable personality disorder). Truncation was used in search terms to capture variations in terminology.

### Eligibility and inclusion criteria

Studies were eligible for analysis if they met the following criteria: a) Research focusing on individuals with features or diagnosis of BPD and community populations endorsing features of BPD that b) contain novel empirical data (quantitative, qualitative, or mixed methods, excluding systematic reviews and case studies), c) are peer-reviewed, d) discuss findings related to chronic emptiness or a related construct in their results or discussion, and e) meet quality assessment.

Due to the limited nature of the research on chronic feelings of emptiness, eligible studies were not restricted by intervention type, comparison, or outcomes. Further, there was no time limit set on searches in order to capture early data regarding emptiness. Language was not restricted as translating software was used.

#### Study selection

Articles which did not meet initial screening criteria were excluded. Articles were then screened by title and abstract by two reviewers for inclusion in full-text review. Disagreement on inclusion of articles for screening was resolved by discussion and advice with another reviewer. Following full text screening, articles were further excluded if they a) were unable to be translated and authors could not be contacted, and b) contained keywords which were discussed only in the context of Schema therapy and Schema modes (e.g. lonely child mode).

#### Risk of bias in individual studies

Following the selection of articles for full-text review, quality was assessed using the Mixed Methods Appraisal Tool (MMAT)–Version 2011. The MMAT has good interrater reliability and content has been validated [42–44]. Although the MMAT is yet to be validated in clinical samples, the absence of a gold standard quality assessment for appraisal of observational studies necessitates the use of modified assessments [45]. Two screening questions were asked for all study types prior to further quality assessment; ‘are there clear research questions or objectives?’ and ‘do the collected data address the research questions?’ The observational descriptive quantitative component of the MMAT was used to examine quantitative studies. This encompasses several factors including appropriate sampling methods, justification of methods and acceptable response rates [43]. The qualitative component of the MMAT was also used, which similarly included factors of appropriate sampling and justification of methods, in addition to understanding the context of information and influence of researchers’ on results.

Studies which satisfied all other eligibility criteria were given an overall rating of quality. Quality scores for quantitative studies ranged from a possible zero to eight, while qualitative study scores ranged from zero to six. Studies with a score of four or higher (quantitative studies) and three or higher (qualitative studies) were deemed appropriate for detailed data extraction and synthesis. Two authors independently assessed study quality, and consensus was reached by discussion. To reduce possible bias towards the previous study published by the authors’ which was included in the review, an independent researcher who had not been involved in the previous study assessed all studies for quality.

### Summary measures and synthesis

Following the quality assessment one researcher extracted data from included studies which was independently checked by a second researcher. Information extracted from articles included aims of the study, study design, participant details, measures, and key results. Quantitative and qualitative studies were summarised in tabular form. One researcher thematically analysed the data to identify key themes in relation to each key word.

## Results

### Study selection

**Fig 1:**
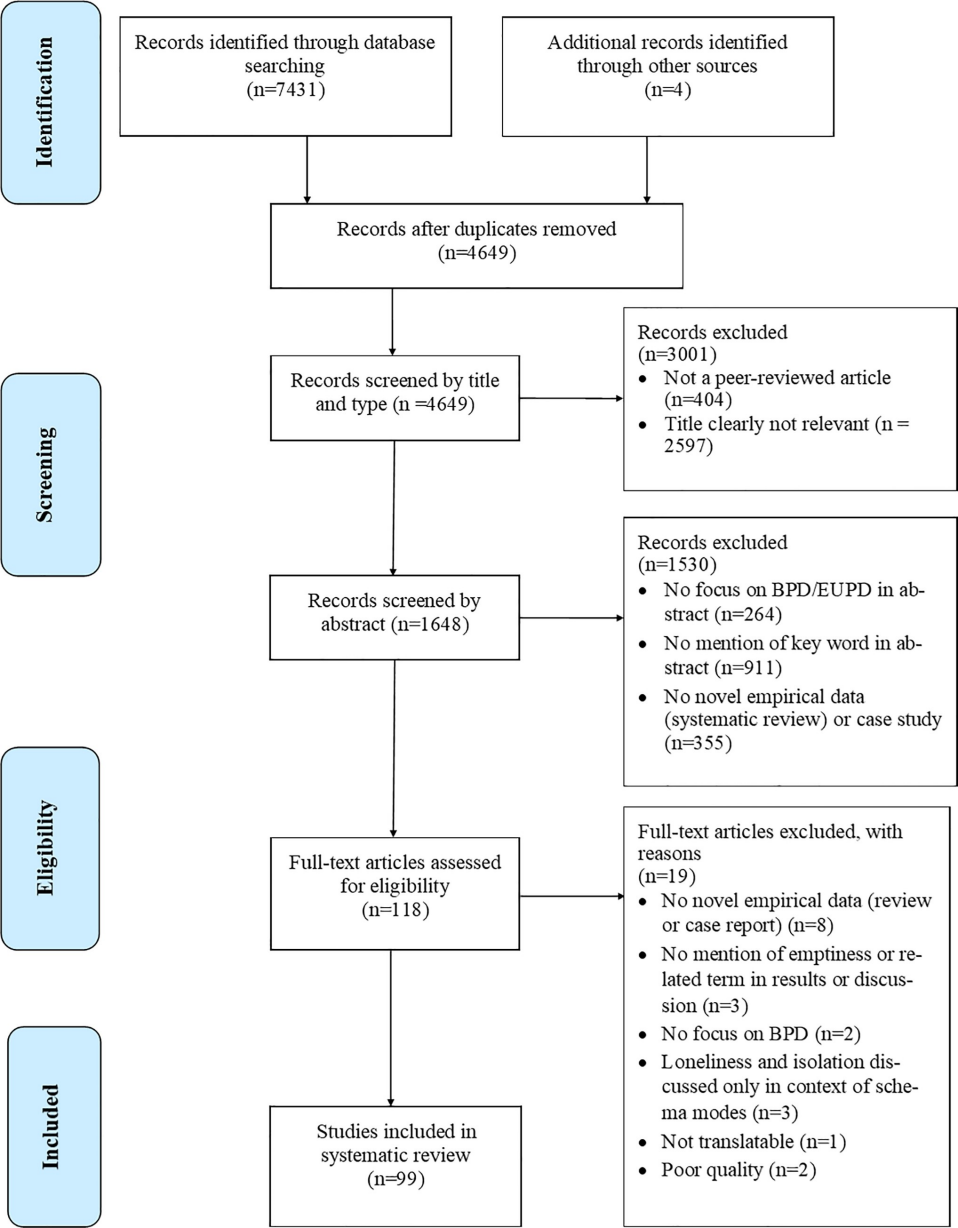
PRISMA flowchart for selection of studies included in systematic review.

A total of 7435 articles were found by electronic database searching (n = 7431) and additional records known to authors (n = 4). Following the removal of duplicates (n = 2786) and exclusion based on article type (n = 404), articles were excluded by title relevance (n = 2597). 1648 article abstracts were screened, and articles were excluded if they had no novel empirical data or were a case study (n = 355), did not have a focus on BPD or Emotionally Unstable Personality Disorder (n = 264), or if there was no mention of emptiness or related keyword in abstract (n = 911). 118 full-text articles were assessed for eligibility. Articles were excluded if they had no novel empirical data (n = 8), no mention of emptiness or related keyword in the results or discussion (n = 3), no focus on BPD (n = 2), if keywords were only used in the context of Schema therapy (n = 3), and if the article was not translatable using software and authors could not be contacted (n = 1). The study selection process is depicted in Fig 1.

Following application of MMAT quality assessment, two studies did not meet quality criteria. One study did not meet screening questions and was excluded from further assessment. The remaining studies (n = 100) were evaluated on the additional four dimensions of the MMAT quantitative descriptive or qualitative tool. One study scored a one and was excluded from further analysis due to low quality. There was a 97.98% agreement between raters for quality assessment; two articles were discussed with a third rater to achieve consensus. All remaining studies (n = 99) scored at least half of the quality criteria and are included in the table of study characteristics, but articles with lower scores should be interpreted with caution (S1 Table).

### Study characteristics

Ninety-nine studies were included in data extraction representing a total of 98,340 participants, with a range of seven to 36,309 participants across individual studies. Eighty-three studies reported on average age of their sample, and the overall average age across studies was 32.1 (*SD* = 11.0). Eighty-seven studies reported on gender ratio within their studies. Participants were predominantly female, with a mean of 77.6% (*SD* = 16.9, range = 36.7–100%). Of the 34 studies reporting participant cultural background, Caucasian participants accounted for an average of 77.2%. Further details of study characteristics are included in Table 1. Studies utilised a wide range of measures to quantify the experience of chronic emptiness and related terms (see Table 2). Table 3 presents a detailed overview of study characteristics.

**Table 1:** Details of included studies.

		Frequency (N)	%
Total studies		99	100
Study design	Quantitative longitudinal	23	23.2
	Quantitative cross sectional	73	73.7
	Qualitative	2	2.0
	Mixed methods	1	1.0
Measure	Measure specific to BPD population ^a^	48	48.5
	General measure used	39	39.4
	Both specific and general measure used	11	11.1
	Unspecified measure	1	1.0
Gender	Both female and male	73	73.7
	Female only	18	18.2
	Male only	1	1.0
	Not specified	7	7.1
Sample type	Outpatients	35	35.4
	Inpatients	29	29.3
	Mixed sample	15	15.2
	Non-clinical sample	10	10.1
	Not specified	10	10.1
Study location	Argentina	1	1.0
	Australia	5	5.1
	Canada	4	4.0
	Denmark	2	2.0
	England	2	2.0
	Finland	1	1.0
	France	3	3.0
	Germany	9	9.1
	Ireland	1	1.0
	Israel	1	1.0
	Italy	2	2.0
	Japan	1	1.0
	Mexico	1	1.0
	Netherlands	1	1.0
	Norway	2	2.0
	South Africa	1	1.0
	Spain	5	5.1
	Switzerland	6	6.1
	United States of America	51	52.5

^a^–Specific measures include developed qualitative questions

**Table 2:** Measures used in selected studies to quantify emptiness or related term and frequency of use.

Measure name	Measure acronym	Frequency (N)
Adult Attachment Projective	AAP	2
Aloneness and Evocative Memory Scale	AEMS	1
Alcohol Use Disorder and Associated Disabilities Interview Schedule-IV	AUDADIS-IV	2
Background Information Schedule	BIS	1
Beck Hopelessness Scale	BHS	26
Bell Object Relations and Reality Testing Inventory	BORRTI	1
Borderline Evaluation of Severity Over Time	BEST	1
Borderline Symptom List	BSL/BSL-23	4
Clinical Global Impression Modified	CGI-M	1
Clinical interview	-	1
Combined Criteria Instrument	CCI	1
Developed measure	-	7
Diagnostic Interview for Borderlines (/Revised)	DIB/DIB-R	18
Diagnostic Interview for Personality Disorders Revised	DIPD-R	2
Experience of Time Alone Scale	ETAS	1
Hurvich Experience Inventory Revised	HEI-R	1
Hopkins Symptom Checklist 90	HSC	2
International Personality Disorders Examination	IPDE	1
McLean Screening Instrument for BPD	MSI-BPD	2
Millon Clinical Multiaxial Inventory	MCMI	1
Multidimensional Personality Questionnaire (/Brief)	MPQ/MPQ-BF	2
Orbach and Mukilincer Mental Pain Scale	OMPP	1
Personality Assessment Interview–Borderline scale	PAI-BOR	1
Personality Diagnostic Questionnaire Revised	PDQ-R	2
Personality Disorder Examination	PDE	2
Personality Inventory for DSM-5	PID-5	1
Rorschach test	-	1
Structured Clinical Interview for DSM-IV Axis II	SCID-II	14
Structured Interview for DSM-IV Personality	SIDP-IV	5
Structured Psychopathological Interview and Rating of the Social Consequences of Psychological Disturbances for Epidemiology	SPIKE	1
Subjective Emptiness Scale	SES	1
Thematic Analysis	-	2
University of California Los Angeles Loneliness Scale	UCLA Loneliness Scale	5
Unspecified measure	-	1
Young Schema Questionnaire	YSQ	1
Zanarini Rating Scale for Borderline Personality Disorder	ZAN-BPD	1

**Table 3:** Characteristics of included studies.

Source	Title	Aim	Participants	Measures	Key results regarding chronic emptiness or related term	Measurement of chronic emptiness or related term
Abela et al., 2003 [46]	Cognitive vulnerability to depression in individuals with borderline personality disorder	Compare cognitive vulnerability to depression in individuals with comorbid BPD and MDD, individuals with MDD only and individuals with neither BPD or MDD.	Parents living in community with history of depressive episode, n = 141 (nNoBPD+NoMDD = 36, nMDD = 89, nBPD+MDD = 16). Median age = 41, 90% female, 84.3% Caucasian.	SCID-I, SCID-II, BDI, BHS, EASQ, DAS, SEQ, RSQ	Individuals with BPD and MDD experienced significantly higher scores of hopelessness compared to MDD only and HC. Individuals with comorbid BPD and MDD displayed significantly greater cognitive vulnerability to depression as measured by hopelessness, low self-esteem, dysfunctional attitudes, and rumination scales.	BHS
Amianto et al., 2011 [47]	Supervised team management, with or without structured psychotherapy, in heavy users of a mental health service with borderline personality disorder: A two-year follow-up preliminary randomised study	Compare the efficacy of Supervised Team Management (STM) and STM plus Sequential Brief Adlerian Psychodynamic Psychotherapy (SB-APP) in BPD treatment.	Individuals engaged in outpatient services in Mental Health Centre in Italy with diagnosis of BPD, n = 35. Mean age = 39.5, 51.4% male. Inclusion criteria: Age 20–50, heavy use of mental health service in prior year, no severe comorbid Axis I disorder, no intellectual, developmental, or cognitive impairment which would impede understanding, no current substance use, no previous psychotherapy intervention.	SCID-I, SCID-II, TCI, SCL-90, STAXI, CGI, GAF, CGI-M, WAI-S	STM and SB-APP was more effective than STM at reducing core psychopathological characteristics including chronic feelings of emptiness. SB-APP may help address emptiness by promoting mentalisation skills, decreasing splitting defenses and increasing tolerance for ambivalence.	CGI-M
Andreasson et al., 2016 [48]	Effectiveness of dialectical behaviour therapy versus collaborative assessment and management of suicidality treatment for reduction of self-harm in adults with borderline personality traits and disorder—a randomized observer-blinded clinical trial	Compare effectiveness of DBT to Collaborative Assessment and Management of Suicidality (CAMS) treatment in reducing self-harm for individuals with BPD symptomology.	Individuals meeting two or more BPD criteria with a recent suicide attempt, n = 108 (nDBT = 57, nCAMS = 51). Mean age = 31.7, 74% female. Inclusion criteria: Age 18–65, no current severe depression, BD, psychosis, anorexia nervosa, substance use, no intellectual, developmental, or cognitive impairment which would impede understanding.	MINI, SCID-II, HDRS-17, presence of self-harm, ZAN-BPD, BDI-II, BSI, BHS, RSE	No significant differences were found between SBT and CAMS for levels of hopelessness in individuals with two or more BPD criteria with a recent suicide attempt.	BHS
Bach, & Sellbom, 2016 [49]	Continuity between DSM-5 Categorical Criteria and Traits Criteria for Borderline Personality Disorder	Examine associations between DSM-5 dichotomous criteria and DSM-5 Section III traits for BPD.	Outpatients from Danish psychiatric service meeting criteria for PD diagnosis, n = 101. Mean age = 29, 68% female.	SCID-II, PID-5	The symptom chronic feelings of emptiness was not significantly correlated with any Section III traits, and only weakly associated with Depressivity. The lack of associations may indicate chronic feelings of emptiness are captured by the personality functioning criteria of Section III conceptualisation.	SCID-II, PID-5
Becker et al., 2006 [50]	Exploratory factors analysis of borderline personality disorder criteria in hospitalised adolescents	Explore factor structure of BPD in hospitalised adolescents meeting criteria for DSM-III-R BPD.	Inpatients at the Adolescent Inpatient Unit at Yale Psychiatric Institute meeting criteria for BPD, n = 123. Mean age = 15.9, 54% male.	SADS, PDE	A four factor solution accounted for 67% of variance. Factor 1 included suicidal threats or gestures and emptiness or boredom. This factor may represent two aspects of dysregulation: the psychological process (emptiness) and maladaptive attempts to relieve tension of this process (suicidal behaviours).	PDE
Bell et al., 1988 [51]	Do object relations deficits distinguish BPD from other diagnostic groups?	Use the Bell Object Relations (OR) Inventory to determine if there is a pattern of OR deficits for individuals with BPD, cross validate this OR profile with a second sample of BPD and compare BPD subjects with other diagnostic samples on OR.	Sample 1: Inpatients at Veterans Administration Medical Centre meeting criteria for BPD diagnosis, n = 44. Mean age = 37.7, 93% male. Sample 2 (cross validation sample): Outpatients meeting criteria for BPD but not any Axis 1 diagnoses, n = 24. Mean age = 30.8, 92% female. Sample 3 (other diagnostic group): Inpatients meeting criteria for schizophrenia, major affective disorder, or schizo-affective disorders but no diagnosis of BPD, n = 82. Mean age = 33, 89% male.	SADS, RDC, New Haven Schizophrenia Index, International Pilot Study for Schizophrenia criteria, Feighner criteria, BORRTI	Individuals with BPD (either inpatient or outpatient) were most identifiable by elevated scores on the Alienation subscale of Bell OR Inventory. Based on only Alienation scores, individuals with BPD could be distinguished from other diagnostic groups with 77–82% predictive accuracy. The internal experience of alienation, lack of intimacy, and loss of trust in interpersonal relations is a common feature of BPD.	BORRTI
Benazzi, 2006 [52]	Borderline personality—bipolar spectrum relationship	Identify which criteria of BPD are related to Bipolar II.	Outpatients with diagnoses of Bipolar II or MDD (in remission) who were further assessed for BPD traits, n = 209. nBD-II = 138, mean age = 39, 77% female. nMDD = 71, mean age = 39, 61% female.	SCID-CV, SCID-II	BPD traits were more common in individuals diagnosed with Bipolar II. Factor analysis of BPD traits found two factors. The first 'affective instability' factor included unstable mood, identity and interpersonal relationships, chronic emptiness, and feelings of anger.	SCID-II
Berk et al., 2007 [53]	Characteristics of recent suicide attempters with and without borderline personality disorder	Identify pathology associated with suicide attempts for individuals with BPD and compare to those with a recent suicide attempt without a BPD diagnosis.	Individuals presenting to hospital due to suicide attempt, n = 180 (nBPD = 65, nNoBPD = 115). Mean age = 34, 57% female, 63% African American. Inclusion criteria: Age 16+, no intellectual, developmental, or cognitive impairment which would impede understanding.	SCID-I, SCID-II, GAF, HAM-D, SSI, SIS, Lethality Scale, BDI-II, BHS, SPSI-R, Psychiatric History Form	Suicide attempters with BPD had higher severity of hopelessness compared to those without BPD.	BHS
Bernheim et al., 2018 [54]	Change of attachment characteristics during dialectic behavioural therapy for borderline patients	Determine if attachment characteristics for individuals with BPD change following DBT.	Individuals with BPD and healthy controls, n = 52 (nBPD = 26, nHC = 26). Inclusion criteria: No intellectual, developmental or cognitive impairment which would impede understanding, no psychosis.	AAP, ASQ, SCID-II, BPI, MWT-B	Individuals with BPD demonstrated more traumatic-dysregulating markers in AAP narratives in response to monadic pictures which may induce feelings of loneliness.	AAP
Bhar et al., 2008 [55]	Dysfunctional beliefs and psychopathology in borderline personality disorder	Examine factor structure of PBQ-BPD in individuals with BPD, understand how factors of PBQ-BPD relate to psychopathology.	Outpatients, inpatients and research participants with diagnosis of BPD, n = 184. Mean age = 33.1, 75.4% female, 55.2% Caucasian. Inclusion criteria: Age 18+, no psychosis.	PBQ-BPD, BDI, SSI, BHS, SCID-I, SCID-II, DIPD-IV	The 'interpersonal distrust' factor of PBQ-BPD correlated with hopelessness, depression, and suicide ideation. The 'dependency' factor was correlated with depression and hopelessness.	BHS
Black et al., 2018 [56]	STEPPS treatment programme for borderline personality disorder: Which scale items improve? An item-level analysis	Determine which items of BEST and ZAN-BPD improve during STEPPS treatment.	Participants in an RCT evaluating STEPPS treatment with diagnosis of BPD and participants in the Iowa correctional system completing STEPPS treatment, n = 193. 81.9% female. No intellectual, developmental, or cognitive impairment which would impede understanding, no psychosis, no substance use disorder.	SCID-I, SCID-II, BEST, ZAN-BPD	Chronic feelings of emptiness significantly improved following STEPPS treatment.	ZAN-BPD, BEST
Bohus et al., 2007 [57]	Psychometric properties of the borderline symptom list (BSL)	Summarise validity, reliability and sensitivity to change for BSL.	Participants from six different samples; inpatient and outpatient females with BPD, male patients with BPD, HCs, participants with other mental disorders, female patients with BPD in inpatient DBT treatment, n = 930. Minimum 53.7% female, 30.4% unreported gender.	BSL, IPDE, MINI, BDI, HAM-D, STAI, STAXI, DES, SCL-90-R	Factor analysis of BSL showed a seven factor solution including a subscale of loneliness.	BSL
Bohus et al., 2001 [58]	Development of the Borderline Symptom List	Develop a self-assessment scale to quantify specific experiences of people with BPD.	Participants with a diagnosis of BPD, n = 308. Mean age = 30.3, 100% women.	99 item early version of BSL	The fifth factor of the symptom list included experiences of social isolation. Items in this factor included 'I believed that nobody understood me', 'I felt isolated from others', 'I felt abandoned by others'.	BSL
Bornovalova et al., 2006 [59]	Temperamental and environmental risk factors for borderline personality disorder among inner-city substance users in residential treatment	Understand temperamental and environmental factors uniquely associated with BPD.	Inpatients in drug and alcohol abuse treatment centre, n = 93. Mean age = 41.5, 56% male, 92.5% African American.	Demographics, MPQ-BF, CTQ-SF, SCID-II.	Results indicate that diagnosis of BPD was associated with several interpersonal factors of temperament including higher rates of alienation.	MPQ-BF
Brickman et al., 2014 [39]	The relationships between non-suicidal self-injury and borderline personality disorder symptoms in a college sample	Understand relationship between BPD factors and symptoms and non-suicidal self-injury in a college sample.	Undergraduate students with and without history of NSSI, n = 724. Mean age = 21.2, 61.2% female, 59.3% Caucasian.	FAFSI, MSI-BPD	Endorsement of disturbed relatedness (chronic emptiness, identity disturbance) was independently associated with history of NSSI. Feelings of chronic emptiness may precede NSSI and may act as motivation to engage in NSSI behaviours in young adults.	MSI-BPD
Brown et al., 2004 [60]	An open clinical trial of cognitive therapy for borderline personality disorder	Identify if cognitive therapy alters risk factors for suicide in clients with BPD.	Individuals reporting suicide ideation or self-harm behaviours in past two months meeting criteria for PD, n = 32. Mean age = 29, 88% female, 72% Caucasian. Inclusion criteria: No psychosis, no intellectual, developmental, or cognitive impairment which would impede understanding.	SCID-I, SCID-II, SSI, HRSD, BDI-II, BHS, PHI, PBQ	Individuals with BPD who receive cognitive therapy experienced a decrease in levels of hopelessness at the end of treatment which was maintained at 18 months follow-up.	BHS
Buchheim et al., 2008 [61]	Neural correlates of attachment trauma in borderline personality disorder: A functional magnetic resonance imaging study	Analyse neural activation patterns of attachment trauma for individuals with BPD, investigating response to stories associated with loneliness and abandonment in the AAP.	Inpatients with diagnosis of BPD and control participants, n = 28 (nBPD = 11, nControl = 17). Mean age = 28.1, 100% female. Inclusion criteria: No serious medical or neurological illnesses (BP, PTSD, DD), no current depressive episode, substance dependence, left-handedness, metal in body, or language difficulties.	SCID-I, SCID-II, DES, BIS, AAP fMRI	Neural differences were found between groups for dyadic pictures, with BPD group showing higher activation of right superior temporal sulcus and lower activation of right parahippocampal gyrus compared to controls. This provides support for the existence of a neural mechanism related to intolerance of aloneness in BPD.	AAP
Chabrol et al., 2001 [62]	Symptomatology of DSM-IV borderline personality disorder in a non-clinical sample of adolescents: Study of 35 borderline cases	Examine symptoms of BPD in a non-clinical adolescent population.	Adolescents willing to complete a personality disorder interview, n = 107. Mean age = 16.7, 68.2% female.	DIB-R, MINI	Chronic feelings of emptiness were experienced by 57.1% of the sample.	DIB-R
Chabrol et al., 2002 [63]	Factor analyses of the DIB-R in adolescents	Examine factor structure of DIB-R in adolescent population.	High school students, n = 118. Mean age = 16.7, 66.9% female.	DIB-R	The first factor of DIB-R, explaining 21% of variance, included painful affect and defenses. Items included loneliness/emptiness, helplessness/hopelessness, depression, anxiety, odd thinking/unusual perceptive experiences, quasi-psychotic experiences.	DIB-R
Chapman et al., 2005 [64]	Factors associated with suicide attempts in female inmates: The hegemony of hopelessness	Examine associations between risk and protective factors with suicide attempts in female inmates.	Female inmates, n = 105. Mean age = 33.9, 100% female, 71.4% Caucasian. No current psychosis or serious reading difficulties.	Demographics, LPC-2, SCID-II, TAAD, BDI-II, BHS, CTQ, RFL, COPE	Hopelessness may mediate the relationship between risk factors (including BPD) and suicide attempts.	BHS
Choi-Kain et al., 2010 [65]	A longitudinal study of the 10-year course of interpersonal features in borderline personality disorder	Determine time to remission of interpersonal BPD symptoms over ten years of follow-up.	Inpatients at McLean Hospital meeting criteria for BPD or another PD, n = 309 (nBPD = 249, nOtherPD = 60). Mean age = 27, 77.1% female, 87% Caucasian. No historical or current schizophrenia, schizoaffective or BP, no intellectual, developmental, or cognitive impairment which would impede understanding (IQ < 70), no organic disorders which could cause psychiatric symptoms, no language difficulties.	SCID-I, DIB-R, DIPD-R	Most interpersonal symptoms of BPD (including intolerance of aloneness) remit significantly over time. The symptom 'affective consequences when alone' declined less substantially over ten years than most other symptoms and was the last interpersonal feature of BPD to remit.	DIB-R, DIPD-R
Conte et al., 1980 [66]	A self-report borderline scale: Discriminative validity and preliminary norms	Develop a self-report measure of BPD and report on psychometric properties.	Participants from four samples, n = 141. HC (n = 50, mean age = 33), outpatients with MDD (n = 36, mean age = 35), outpatients with BPD (n = 35, mean age = 33), and inpatients with Schizophrenia (n = 20, mean age = 32).	BSI	Items related to feelings of chronic emptiness discriminated the BPD group from all other groups.	BSI
Cottraux et al., 2009 [67]	Cognitive therapy versus Rogerian supportive therapy in borderline personality disorder	Compare cognitive therapy to Rogerian supportive therapy over one year for individuals with BPD.	Outpatients with diagnosis of BPD, n = 65. Mean age = 33.5, 76.9% female. Inclusion criteria: Age 18–60, no psychosis, no substance use disorder, no antisocial behaviours.	MINI, DIB-R, CGI, HDRS, BDI, BHS, BAI, YSQ-II, IVE, SHBCL, TRES, SAS	Hopelessness improved more in cognitive therapy compared to Rogerian supportive therapy.	BHS
Ellison et al., 2016 [37]	The clinical significance of single features of borderline personality disorder: Anger, affective instability, impulsivity, and chronic emptiness in psychiatric outpatients	Understand which DSM-5 BPD criteria are associated with psychosocial morbidity in outpatients.	Individuals presenting to Rhode Island Hospital for outpatient psychiatric care reporting either no BPD symptoms, affective instability, emptiness, or impulsivity symptoms, n = 1870 (nNoBPD = 1387, nImpulsivity = 114, nAffectiveInstability = 86, nEmptiness = 170, nAngerOnly = 113). Mean age = 38, 60% female, 92% Caucasian. Inclusion criteria: Age 18+, no intellectual, developmental or cognitive impairment which would impede understanding, no difficulty communicating in English.	SIDP-IV, SCID-I, GAF, items from SADS (current suicidality, current social function, prior psychiatric hospitalisations, history of suicide attempts)	Participants experiencing one BPD symptom had higher rates of comorbid mood disorder and lower functioning compared to participants without BPD symptoms. Impulsivity and emptiness groups had poorer work function and emptiness and anger groups had lower social function than no BPD group. Emptiness group had poorer psychosocial function compared to group without BPD criteria on all measures.	SIDP-IV
Espinosa et al., 2009 [68]	Risk and suicide lethality in patients with borderline personality disorder in a psychiatric hospital	Assess suicidal risk and lethality in individuals with BPD.	Individuals with BPD who presented to hospital with suicidal ideation or following suicide attempt, n = 15. Mean age = 29.5, 94% female.	SCID-II, SIS, BHS, DSQ, RRS	Almost half of the sample of individuals with BPD endorsed severe hopelessness after presenting to hospital for suicide attempt or ideation.	BHS
Fertuck et al., 2016 [69]	The specificity of mental pain in borderline personality disorder compared to depressive disorders and healthy controls	Clarify the differences in mental pain between a BPD group, a depressed group, and healthy controls. Identify subtypes of mental pain common to each group.	Individuals involved in ongoing hospital clinical research meeting diagnosis of BPD or DD or recruited through community sampling, n = 110 (nBPD = 57, nDD = 22, nHC = 31). Mean age = 31, 75% female, 44% Caucasian. Inclusion criteria: Age 18–55, BPD and DD groups: no history of psychotic or neurological disorder, no other medical or psychological condition. HC group: no current or past psychological disorder.	SCID-I, SCID-II, OMPP, BDI, HAM-D	BPD group had significantly higher depression and hopelessness, BPD and DD groups had significantly higher ratings of emptiness compared to HC.	SCID II, OMPP
Flynn et al., 2017 [70]	Standard 12 month dialectical behaviour therapy for adults with borderline personality disorder in a public community mental health setting	Evaluate the use of DBT for BPD in community mental health and determine outcomes following DBT.	Outpatients with BPD seeking community mental health treatment, n = 71. Mean age = 40, 85.9% female.	BSL-23, BAI, BHS, BSS, BDI-II, WHOQOL-BREF	DBT was associated with significant reduction in hopelessness.	BHS
Fritsch et al., 2000 [71]	Personality characteristics of adolescent suicide attempters	Examine personality disorder symptoms and their relationship to hopelessness in adolescents with a suicide attempt.	Inpatient adolescents who had attempted suicide, n = 137. Mean age = 15.1, 80.3% female, 76% Caucasian. Inclusion criteria: Age 13–18.	MAPI, DIB-R, HSC	Individuals with higher scores of hopelessness scored higher on Inhibited and Sensitive scales of MAPI and more dysfunctional scores on Affect Regulation scale of DIB-R. Adolescents with high hopelessness had a negative sense of self in most factors of personality function.	HSC
Garcia-Alandete et al., 2014 [72]	Predicting role of the meaning in life on depression, hopelessness, and suicide risk among borderline personality disorder patients	Understand the relationship between meaning in life and depression, hopelessness, and suicidality in a BPD sample.	Individuals from public mental health service meeting criteria for BPD diagnosis, n = 80. Mean age = 32, 93% female, 100% Caucasian. Inclusion criteria: Age 16–60, no psychotic disorder, no intellectual, developmental or cognitive impairment which would impede understanding, no difficulty communicating in Spanish.	PIL-10, BDI-II, BHS, SRS, SCID-II, SCID-I	Meaning in life was a significant negative predictor of depression, hopelessness, and suicide risk.	SCID II
Glenn & Klonsky, 2013 [73]	Nonsuicidal self-injury disorder: An empirical investigation in adolescent psychiatric patients	Identify if NSSI occurs without BPD and whether it indicates significant impairment beyond a diagnosis of BPD.	Adolescent psychiatric inpatients, n = 198. Mean age = 15.1, 74% female, 64% Caucasian. Inclusion criteria: No psychosis, aggressive or severe suicide-related behaviours, no intellectual, developmental, or cognitive impairment which would impede understanding.	ISAS, SCID-II, MINI-Kid, DERS, UCLA Loneliness Scale.	Adolescents with NSSI disorder had higher rates of loneliness and emotion dysregulation compared to adolescents without NSSI disorder.	UCLA Loneliness scale
Goodman et al., 2013 [74]	Developmental trajectories to male borderline personality disorder	Identify traits and symptoms of male children who develop BPD through parental survey.	Parents of offspring with and without BPD, n = 263. Mean age = 53.9, 93% female. Inclusion criteria: Male gendered offspring.	MSI-BPD	Endorsement of feelings of chronic emptiness had the greatest discrepancies between adult male children with BPD (97%) and adult male children without BPD (8%). By childhood, 41% of males with later BPD experienced emptiness versus 1% of male children without BPD. In adolescence, 59% of males with later BPD endorsed emptiness compared to 4% of males without BPD.	MSI-BPD
Harford et al., 2018 [75]	Borderline personality disorder and violence toward self and others: A national study	Identify BPD criteria which are related to violence towards self and others.	Participants in the NESARC-III study without BPD, with subthreshold BPD and with BPD, n = 36309 (nSubthresholdBPD = 19404, nBPD = 4301). Mean age = 45.5.	NESARC-III suicide attempt and violence questions, AUDADIS-IV	In the total population, symptoms of emptiness, abandonment fear, self-harm, and intense anger all characterised violence towards self (suicide attempts). In the BPD population, the presence of emptiness, self-harm, impulsivity and anger created higher odds for violence towards self and others versus no violence.	AUDADIS-IV
Hauschild et al., 2018 [76]	Behavioural mimicry and loneliness in borderline personality disorder	Determine if behavioural mimicry is altered in BPD compared to healthy controls and if level of mimicry is linked to feelings of loneliness.	Participants with BPD and healthy controls, n = 51 (nBPD = 26, nHC = 25). Mean age = 29.4, 100% female. Inclusion criteria: No left-handedness, no psychosis or BP disorder, no current substance use, history of organic brain disease, brain damage, or neurological illness. No current pregnancy. Healthy controls: No current or lifetime psychiatric diagnoses.	SCID-I, IPDE, BSL, UCLA Loneliness scale, finger-tapping task	Individuals with BPD reported higher levels of loneliness compared to HC. Behavioural mimicry was lowest in individuals with BPD with the highest loneliness scores, suggesting behavioural imitation becomes disengaged or the motivation to engage with others is reduced when people with BPD experience high levels of loneliness.	UCLA Loneliness scale
Hengartner et al., 2014 [77]	Interpersonal functioning deficits in association with DSM-IV personality disorder dimensions	Expand literature on interpersonal function for PD focusing on social functioning.	Swiss individuals representative of general population, n = 511. 55.6% female. Inclusion criteria: Age 20–41.	SCL-27, ADP-IV, SPIKE	BPD was more highly associated with feelings of loneliness compared to other PDs.	SPIKE
Hoertel et al., 2014 [78]	Examining sex differences in DSM-IV borderline personality disorder symptom expression using Item Response Theory (IRT)	Identify sex differences for reporting of BPD criteria in a general population sample and BPD subsample using item response theory.	USA individuals participating in second wave of NESARC both with and without BPD, n = 34 481 (nBPD subsample = 1030). General sample mean age = 49, 57.9% female, Caucasian 70.7%. BPD sample mean age = 39.8, 62.5% female, Caucasian 71.7%. Inclusion criteria: Age 18+, no participants outside USA or on active military duty.	AUDADIS-IV	Prevalence of chronic feelings of emptiness was significantly higher in females compared to males in both BPD subsample and general population.	AUDADIS-IV
Horesh et al., 2003 [79]	Comparison of the suicidal behaviour of adolescent inpatients with borderline personality disorder and major depression	Determine if there is a difference in emotional motivation between BPD and MDD recent adolescent suicide attempters.	Adolescents admitted to psychiatric unit meeting diagnosis for BPD or MDD and recent or no prior suicide attempts, n = 65 (nBPD = 33, nMDD = 32). 77% female, 100% Jewish heritage from lower-middle SES. Inclusion criteria: Age 13–18, no substance use disorder, no intellectual, developmental or cognitive impairment which would impede understanding, fluency in Hebrew.	CSPS, BDI, BHS, MAI, OAS, ICS, SIS, SADS, DIB-R	For BPD and MDD groups no differences on scores of depression and hopelessness were found. For recent suicide attempters compared to non-suicidal group hopelessness was higher for recently suicidal adolescents. Depression and hopelessness were associated with suicidal behaviour in both BPD and MDD groups.	DIB, BHS
Hulbert & Thomas, 2007 [80]	Public sector group treatment for severe personality disorder: a 12-month follow-up study	Evaluate a treatment program for individuals with BPD with a history of unsuccessful treatments and severe self-harm after 12 months.	Female Anglo-Australian individuals receiving Spectrum Group Treatment who had a diagnosis of PD with a history of unsuccessful mental health treatment and current self-harm, n = 27. Mean age = 34, 100% female. Inclusion criteria: Age 16–64, no acute psychiatric disorders or limited English.	SCID-I, SCID-II, BAI, BDI, BHS, DES, PHI, WCCL, WHOQOL-BREF	Following Spectrum Group Treatment Program, clinically significant gains in reported levels of hopelessness were found.	BHS
James et al., 1995 [81]	Borderline personality disorder: A study in adolescence	Determine presentation and symptom experience of adolescents with BPD compared to psychiatric controls, and identify family predictors.	Adolescents admitted to Oxford Regional Adolescent Unit over two years, n = 48 (nBPD = 24, nControl = 24). Mean age = 14.9, 83% female. Inclusion criteria: No intellectual, developmental or cognitive impairment which would impede understanding.	DIB, chart and case notes, GAS	Adolescents with BPD experienced high levels of boredom and anhedonia which resulted in dysthymia or depressive experiences.	DIB
Javaras et al., 2017 [82]	Functional outcomes in community-based adults with borderline personality disorder	Compare levels of functional impairment between individuals with BPD in clinical treatment programs, individuals with BPD in the community and individuals without BPD.	Probands with and without BPD in general community or clinical treatment programs and their relatives, n = 1127 (nBPD = 225 [clinical = 61, community = 164], nNoBPD = 902), Proband age 18–35, 100% female.	DIPD-IV, DIB-R, BIS	Individuals with BPD in clinical treatment programs were more likely to experience higher levels of social isolation compared to individuals with BPD in the community.	BIS
Johansen et al., 2004 [83]	An investigation of the prototype validity of the borderline DSM-IV construct	Evaluate prototype validity of construct of BPD in DSM-IV.	Individuals engaged in day treatment programs in Norway with PD, n = 930. Mean age = 34.6, 72% female.	SCID-II, MINI, GAF, SCL-90,	Chronic feelings of emptiness had the lowest correlation with other BPD criteria. It also had the weakest correlation with a BPD diagnosis. Individuals who endorsed the emptiness criterion scored significantly higher on measures of depression. One reason for the low correlations may be the absence of an operational definition of emptiness which indicates a need for a definition of the emptiness criterion.	SCID-II
Kerr et al., 2018 [84]	Depression and substance use disorders in the offspring of depressed parents as a function of the parent's borderline personality disorder symptomatology	Identify risk of MDD and substance use disorder in children of psychiatric outpatients with both MDD and BPD features.	Outpatients at Rhode Island Hospital and their children, n = 2923 (nParents = 912, nOffspring = 2011). Parent group mean age = 45.2, 68.3% female, 83.9% Caucasian. Offspring group mean age = 19.6, 51.5% male.	SCID-I, SIDP-IV	Of BPD criteria chronic feelings of emptiness had the highest endorsement among parents at 30% of the sample. Children of parents with chronic emptiness were at significantly higher risk of developing substance use disorders compared to children of parents without feelings of emptiness. Feelings of emptiness reported by the parent predicted offspring substance use even after controlling for other BPD criteria.	SIDP-IV
Klonsky, 2008 [85]	What is emptiness? Clarifying the 7th criterion for borderline personality disorder	Define meaning and clinical significance of the BPD criterion chronic feelings of emptiness in a sample of college students.	Study 1—College students with five or more historical instances of non-suicidal self-injury, n = 45. Mean age = 19.4, 78% female, 89% Caucasian. Study 2—College students in undergraduate psychology courses, n = 274. Mean age = 19, 53% female, 38% Caucasian, 38% Asian.	Analysis 1: structured interview of affect-states (developed item). Analysis 2: MSI-BPD, YRBS, DASS-21	Study 1: 67% participants reported feeling empty before self-harm behaviours. Correlations were low between affect states of emptiness and boredom. High correlations were found between emptiness and affect states of hopelessness, isolation, and loneliness before and after self-harm. Study 2: Emptiness had a moderate correlation with depression and anxiety. Excluding the suicidal criterion, the criterion of chronic emptiness showed the strongest association with history of suicidal ideation.	MSI-BPD, How often do you feel empty before and after self-injury?
Koons et al., 2001 [86]	Efficacy of dialectical behaviour therapy in women veterans with borderline personality disorder	Compare DBT treatment to treatment as usual.	Outpatient female veterans meeting diagnostic criteria for BPD in treatment for DBT and TAU, n = 20 (nDBT = 10, nTAU = 10). Mean age = 35, 100% female, 75% Caucasian.	SCID-II, SCID-I, PHI, BSI, BHS, BDI, HAM-D, HARS, SAES, DES	Hopelessness improved significantly more in DBT compared to TAU.	BHS
Lenzenweger et al., 2012 [87]	Exploring the interface of neurobehaviourally linked personality dimensions and personality organization in borderline personality disorder: The Multidimensional Personality Questionnaire and Inventory of Personality Organization	Examine relationships between psychometric indicators of neurobehavioural and psychodynamic processes in BPD.	Individuals with a diagnosis of BPD, n = 92. Mean age = 30.7, 93.5% female.	IPDE, SCID-I, IPO, MPQ	Alienation (negative emotionality) was significantly associated with identity diffusion, primitive defences and reality testing.	MPQ
Leppänen et al., 2016 [88]	Association of parasuicidal behaviour to early maladaptive schemas and schema modes in patients with BPD: The Oulu BPD study	Identify if there are specific early maladaptive schemas or schema modes that are linked with parasuicidal behaviour in BPD.	Individuals with BPD and severe symptoms of previous unsuccessful treatment, n = 60. Mean age = 32.4, 85% female. Inclusion criteria: Age 20+, no psychosis, BP or substance use disorder.	BPDSI-IV, SCID-II, SCID-I, YSQ, YAMI	BPD individuals with parasuicidal behaviour demonstrated higher scores on social isolation/alienation schema compared to BPD individuals without parasuicidal behaviour.	SCID-II, YSQ
Liebke et al., 2017 [89]	Loneliness, social networks, and social functioning in borderline personality disorder	Investigate social isolation and social functioning in relation to loneliness in BPD. Identify if loneliness is a unique factor or if it is accounted for by isolation and impaired functioning.	Individuals with BPD and healthy controls recruited by German Research Foundation, n = 80 (BPD = 40, HC = 40). Mean age = 27, 100% female. BPD group inclusion criteria: No psychosis or BP, current substance use, current pregnancy, history of organic brain disease, brain damage or neurological disorder. HC group inclusion criteria: No psychiatric diagnoses.	BSL-23, ZAN-BPD, IPDE, UCLA Loneliness Scale, SNI, SFS, GAF	BPD group reported higher levels of loneliness compared to HC. Individuals with BPD had smaller and less diverse social networks, and poorer social/interpersonal function which were linked to increased loneliness. After controlling for social-cognitive deficits, the BPD group still had higher loneliness scores, suggesting there are other factors which contribute to feelings of loneliness.	UCLA loneliness scale
Marco et al., 2014 [90]	The meaning in life as mediating variable between depression and hopelessness in patients with borderline personality disorder	Analyse the mediating role of meaning in life between depression and hopelessness for people with BPD.	Participants with BPD from mental health services in Spain, n = 80. Mean age = 32.3, 93% female. Inclusion criteria: Age 16–60, no psychosis, no intellectual, developmental or cognitive impairment which would impede understanding.	SCID-I, SCID-II, BDI-II, PIL, BHS.	Meaning in life mediated the relationship between depression and hopelessness. A greater meaning in life was associated with less hopelessness.	BHS
Marco et al., 2015 [91]	Meaning in life and non-suicidal self-injury: A follow-up study with participants with borderline personality disorder	Identify if there is a link between low meaning in life and self-harm in participants with BPD at intake time point. Indicate if there is a relationship between low meaning in life and depression, hopelessness and self-harm at follow-up. Understand predictors of self-harm frequency over time.	Individuals engaged in outpatient program who met criteria for BPD, n = 80. Mean age = 32, 94% female, 100% Caucasian. Inclusion criteria: Age 16–60, no psychosis, no intellectual, developmental or cognitive impairment which would impede understanding, fluent in Spanish.	SCID I, SCID II, relevant clinical information inventory (developed items for frequency of self-harm), PIL-10, BHS, BDI-II	Individuals scoring low on meaning in life measures at baseline had higher frequency of self-harm and severe levels of depression and hopelessness compared to those with high meaning in life. Over twelve months meaning in life was negatively correlated with self-harm frequency, hopelessness, and depression.	SCID II, BHS
Marco et al., 2017 [92]	The buffer role of meaning in life in hopelessness in women with borderline personality disorders	Extend on previous findings in a clinical sample to explore the effect of meaning in life on the relationships between previous suicide attempts and hopelessness.	Individuals engaged in outpatient program for BPD, n = 124. Mean age = 31, 100% female. Inclusion criteria: Age 13–56, no psychosis, no intellectual, developmental or cognitive impairment which would impede understanding, fluent in Spanish.	SCID II, PIL-10, BHS, SRS	Meaning in life moderated the relationship between suicide risk factors (previous attempts) and hopelessness. Higher scores of meaning in life reduced the effect of risk factors on hopelessness.	SCID II, BHS
McGlashan, 1987 [93]	Testing DSM-III symptom criteria for schizotypal and borderline personality disorders	Identify which individual symptoms are most discriminating between BPD and SPD.	Individuals in the Chestnut Lodge Follow-Up Study with diagnosis of BPD, SPD or comorbid BPD and SPD, n = 109 (nBPD = 81, nSPD = 10, nBPD+SPD = 18). Inclusion criteria: No psychosis or BP disorder.	DIB	The least discriminating BPD criteria were intolerance of aloneness and anger.	DIB
McQuillan et al., 2005 [94]	Intensive dialectical behaviour therapy for outpatients with borderline personality disorder who are in crisis	Assess modified intensive DBT program on outcomes of hopelessness, depression, and social function.	Outpatients with diagnosis of BPD who identified as being in crisis, n = 127. Mean age = 30.7, 81% female. Inclusion criteria: No psychosis, BP disorder, developmental disorder, substance use disorder or eating disorder.	IPDE, DBI, BHS, SASS	Significant improvements in hopelessness and depression were found after modified intensive DBT treatment.	BHS
Meares et al., 2011 [95]	Is self disturbance the core of borderline personality disorder? An outcome study of borderline personality disorder factors	Determine the core disturbance in BPD which endures over time in relation to Clarkin's three factor model.	Individuals with a BPD diagnosis who received either one year of conversational model (CM) therapy or treatment as usual (TAU), n = 60 (nCM = 29, nTAU = 31). Mean age = 29, 55% female.	WSS, SDS	The constellation of symptoms relating to self (emptiness, identity disturbance, fears of abandonment, interpersonal difficulties) are more chronic than symptoms relating to regulation and may reflect the core problem of BPD. Therapeutic treatment may address these symptoms.	Unspecified
Miller et al., 2018 [38]	A 1-year follow-up study of capacity to love and work: What components of borderline personality disorder most impair interpersonal and vocational functioning?	Examine symptoms of BPD and their influence on psychosocial function over 12 months in a BPD sample.	Patients presenting to mental health services for treatment of personality disorder, n = 199. Mean age = 32.3, 72.9% female.	GAF, SOFAS, WHODAS 2.0, BPD symptom severity (developed)	Severity of chronic emptiness, identity disturbance, mood dysregulation, impulsivity, and self-harm at intake predicted impaired work function at 12 months follow-up. Mediation modelling found a significant relationship between severity of chronic emptiness (intake) and days out of work (follow-up), which was mediated by severity of impulsivity and frequency of self-harm at intake. Chronic feelings of emptiness may underlie and contribute to behavioural symptoms of impulsivity and self-harm.	BPD symptom severity (MSI question)
Miskewicz et al., 2015 [96]	A contingency-oriented approach to understanding borderline personality disorder: Situational triggers and symptoms	Identify proximal symptoms of BPD which trigger symptomology.	Participants with BPD and general population, n = 255 (nBPD = 77, nHC = 178). Mean age = 44, 67.8% female, 60% Caucasian. Inclusion criteria: Age 18–65, no scores below 24 on MMSE, history of violent crimes, current substance use, current psychosis, or actively suicidal participants.	MINI, SIDP-IV, experience sampling method reports	In the BPD sample, presence and severity of BPD symptomology was contingent on situational triggers. Being alone triggered the experiences of all BPD symptoms except self-harm. Further, as severity of BPD increased, so did the intensity of being alone. Increases in symptoms of emptiness, disturbed self-concept, impulsivity, unstable mood, anger, and dissociative experiences were significantly associated with being alone.	emptiness: I felt hollow inside; I had feelings of emptiness
Morgan et al., 2013 [97]	Differences between older and younger adults with borderline personality disorder on clinical presentation and impairment	Compare younger and older individuals with BPD on mood disorder comorbidity, frequency of symptomology, and functionality.	Individuals engaging in outpatient services meeting BPD criteria, n = 143 (nYounger = 97, nOlder = 46), 76% female. Inclusion criteria: Age 18+, no intellectual, developmental or cognitive impairment which would impede understanding, fluent in English.	SIDP-IV, SCID I, SADS, GAF, self-injury questionnaire	Compared to younger adults with BPD, older adults were more likely to endorse chronic emptiness and poorer social functioning than younger adults. Emptiness may be less likely to change over time for individuals with BPD.	SIDP-IV
Morton et al., 2012 [98]	Acceptance and commitment therapy group treatment for symptoms of borderline personality disorder: A public sector pilot study	Report on outcomes of pilot study for group ACT intervention for people with BPD compared to TAU, and investigate what mediates improvement in BPD symptoms, anxiety, depression, stress, and hopelessness.	Outpatients meeting four or more criteria for BPD who were engaged with mental health services, n = 41 (nACT = 21, nTAU = 20). Mean age = 34.8, 92.7% female. Inclusion criteria: No current psychosis, no violent behaviours, no intellectual, developmental, or cognitive impairment which would impede understanding, no difficulty understanding English.	SCID-I, SCID-II, BEST, DASS, BHS, AAQ, FFMQ, ACS, DERS	Severity of hopelessness improved more significantly in the ACT group compared to TAU group. Emotion regulation and acceptance skills mediated the relationship between the ACT group treatment and improvements in hopelessness. This suggests developing emotion regulation skills can reduce hopelessness for people with BPD.	BHS
Nicastro et al., 2016 [99]	Psychometric properties of the French borderline symptom list, short form (BSL-23)	Examine psychometric properties of the French version of BSL-23	Outpatients with diagnoses of BPD or ADHD, n = 310 (nBPD = 265, nADHD = 45). BPD sample mean age = 32.2, 90.2% female.	DIGS, SCID-II, BSL-23, DIVA, WURS, BDI-II, BIS, BHS, STAXI	French BSL-23 was highly correlated with severity of hopelessness.	BSL, BHS
Nisenbaum et al., 2010 [100]	Variability and predictors of negative mood intensity in patients with borderline personality disorder and recurrent suicidal behaviour: Multilevel analyses applied to experience sampling methodology	Identify patterns of variability in mood using EMA over 21 days in a sample of BPD individuals and explore if these patterns can be predicted by risk factors associated with suicidal behaviours.	Outpatients with a diagnosis of BPD had engaged in at least two acts of suicidal behaviour with intent to die, with at least one being in previous two years, n = 82. Mean age = 33.5, 82.9% female. Inclusion criteria: Age 18–65	SCID-II, BIS, BDHI, SWLS, BDI-II, BHS, SSI, SBQ, CTQ	Daily mood ratings were dependent on severity of hopelessness throughout day for individuals with BPD.	BHS
Ntshingila et al., 2016 [101]	Experiences of women living with borderline personality disorder	Explore life experiences of women with BPD in South Africa.	Females with BPD in a psychotherapy ward, n = 8. Mean age = 28, 100% female. Inclusion criteria: Age 18–40	Qualitative question—"Tell me your life story"	An emergent theme of life stories among participants was chronic feelings of emptiness in relation to the self. Specifically, the theme chronic emptiness consisted of subthemes of 'distorted self-image' and 'lack of identity'. Participants showed a sense of worthlessness and powerlessness when discussing these themes, and reportedly filled the 'void' of emptiness by engaging in impulsive behaviours.	Themes from qualitative responses
Nurnberg et al., 1986 [102]	Core criteria for diagnosing borderline patients	Examine diagnostic criteria for individuals with BPD and determine the essential features for diagnosis.	Inpatients with BPD at a university teaching hospital and healthy controls, n = 37 (nBPD = 17, nHC = 20). BPD group age range 17–35, 59% female. Inclusion criteria: No intellectual, developmental or cognitive impairment which would impede understanding. No substance use disorder, no psychosis, no significant medical illness.	DIB	Compared to HCs, the BPD group was characterised by feelings of chronic emptiness, depressive loneliness, and boredom. Chronic emptiness or loneliness was present in 94% of the BPD group compared to 40% of the HC group. Results suggest chronic emptiness/loneliness, impulsivity, unstable relationships, and acting out behaviours are the most common symptoms among the BPD group.	DIB
Nurnberg et al., 1987 [103]	Efficient diagnosis of borderline personality disorder	Identify essential features of BPD and determine how many of DSM-III criteria are necessary for a diagnosis of BPD.	Inpatients with a diagnosis of BPD and healthy controls, n = 37 (nBPD = 17, nHC = 20). 59.5% female. Inclusion criteria: Age 16–45, no current psychosis, no intellectual, developmental, or cognitive impairment which would impede understanding, no substance use disorder, no significant comorbid mental health disorder (BPD group) or psychiatric history (HC group).	DIB, CCI	Chronic feelings of depressive emptiness, loneliness, and boredom, disturbed interpersonal relations, and impulsive behaviours were the most discriminative criteria for BPD participants.	CCI
Nurnberg et al., 1991 [104]	Hierarchy of DSM-III-R criteria efficiency for the diagnosis of borderline personality disorder	Identify discriminating features of BPD and evaluate diagnostic efficiency of DSM-III criteria.	Outpatients with diagnosis of anxiety disorder or other Axis 1 disorder assessed for BPD, n = 110 (nBPD = 22). Mean age = 35, 55% female. Inclusion criteria: No psychosis, major affective disorder, no impairments which would impede understanding, no substance use disorder, must have completed at least one year of psychological treatment.	Clinical interview, SIDP, DIB	Chronic emptiness, boredom and loneliness was the third most discriminating criteria for BPD diagnosis, following interpersonal difficulties and impulsivity.	DIB
Ohshima, 2001 [105]	Borderline personality traits in hysterical neurosis	Compare psychopathology of BPD and hysterical neurosis.	Inpatients and outpatients diagnosed with BPD or hysterical neurosis (dissociative disorder or conversion disorder in DSM-III), n = 88 (nBPD = 48, nHystericalNeurosis = 40). Mean age = 26.1, 67% female. Inclusion criteria: 40 years or younger.	DIB	BPD group showed higher scores of intolerance of aloneness and lower scores of loneliness suggesting both groups experience loneliness but people with BPD find being alone and feeling lonely intolerable.	DIB
Oldham et al., 1996 [106]	Relationship of borderline symptoms to histories of abuse and neglect: A pilot study	Identify whether individuals with BPD who have histories of abuse and neglects can be differentiated from individuals with BPD or other PDs without abuse and neglect.	Patients applying for long-term inpatient treatment for personality disorder, n = 50 (nBPD = 44, nOtherPD = 6).	PDQ-R, Patient history questionnaire	Factor analysis showed abuse history was correlated with chronic emptiness. One subtype of BPD may include a sense of emptiness, relationship instability, and abandonment fears.	PDQ-R
Perez et al., 2014 [107]	Comparison of clinical and demographic characteristics among borderline personality disorder patients with and without suicidal attempts and non-suicidal self-injury behaviours	Explore demographic, clinical, and symptom differences between groups—Individuals with BPD who (a) have engaged in self-harm attempted suicide, (b) have engaged in self-harm only, and (c) engaged in neither self-harm nor attempted suicide.	Individuals engaged in outpatient services for BPD, n = 85. Mean age = 32, 94% female. Inclusion criteria: Age 13–60, no intellectual, developmental or cognitive impairment which would impede understanding, fluent in Spanish.	Clinical information inventory, self-harm history, suicide attempt history (developed items), SCID I, SCID II, BHS, BDI-II, SRS	The self-harm and suicide attempt group had the higher number of prior suicide attempts among groups and had the highest level of hopelessness. Higher levels of hopelessness are associated with more severe suicide-related behaviours.	SCID II, BHS
Perroud et al., 2013 [108]	Response to psychotherapy in borderline personality disorder and methylation status of the BDNF gene	Compare DNA methylation status of BDNF exons I and IV in BPD subjects to control subjects. Determine if epigenetic processes can be changed by psychological treatment of BPD.	Outpatients with BPD attending intensive DBT program and HC group, n = 167 (nBPD = 115, nHC = 52). Mean age = 35.5, 79% female. Inclusion criteria: No participants with suicidal behaviour, severe impulse dyscontrol or severe anger difficulties.	SCID-II, DIGS, BDI-II, BHS, BIS, CTQ	Following intensive DBT, there was a significant decrease in severity of hopelessness. Changes in methylation status of BDNF was significantly associated with change in hopelessness scores.	BHS
Pinto et al., 1996 [109]	Borderline personality disorder in adolescents: Affective and cognitive features	Determine affective and cognitive symptoms of BPD in adolescents, and identify if depressed adolescents with and without BPD can be distinguished.	Females admitted to adolescent inpatient unit at psychiatric hospital, n = 40 (nBPD = 19, nNoBPD = 21). Mean age = 14.9, 100% female. Inclusion criteria: Age 13–17 years, no psychosis or delirium, no intellectual, developmental or cognitive impairment which would impede understanding, English as first language.	DIB-R, DICA-R-A, BDI, RCMAS, STAXI, HSC, LOC, CASQ, PHCSCS	Severity of hopelessness did not distinguish between depressed adolescents with and without BPD, indicating it is not unique to BPD. Depressed adolescents with BPD were distinguishable by poor self-concept, perhaps related to identity disturbance and chronic emptiness.	HSC, DIB-R
Powers et al., 2013 [110]	Symptoms of borderline personality disorder predict interpersonal (but not independent) stressful life events in a community sample of older adults	Examine whether personality pathology predicts dependent and independent stressful life events in older adults.	Community sample engaged in the St Louis Personality and Aging Network Study who had completed baseline and one follow-up, n = 1630. Mean age = 60, 54% female, 69% Caucasian. Inclusion criteria: Age 55–64.	SIDP-IV, LTE-Q, BDI-II	Unstable interpersonal relationships and impulsivity was associated with higher number of stressful life events, and chronic emptiness was associated with less stressful life events.	SIDP-IV
Price et al., 2019 [111]	Subjective emptiness: A clinically significant trans-diagnostic psychopathology construct	Identify core features of emptiness across diagnosis and create a quantitative measure of emptiness.	Sample 1: Undergraduate students, n = 543. Mean age = 20.2, 76.8% female, 44.5% Hispanic/Latino. Sample 2: Adults diagnosed with psychiatric disorders, n = 1067. Mean age = 29.8, 67.1% female, 81.8% Caucasian. Sample 3: Adults diagnosed with psychiatric disorders, n = 1016. Mean age = 27.5, 56.3% female, 81.5% Caucasian. Inclusion criteria: Age 18+, fluent in English.	ZAN-BPD, PIL-SF, BIS-Brief, CES-D 10, SCIM, PID-5-SF, SES	A unidimensional construct of emptiness was found with core features of detachment from self and others, hollowness, aloneness, disconnection, and unfulfillment. The subjective emptiness scale was developed as a transdiagnostic measure of emptiness.	SES
Rebok et al., 2015 [112]	Types of borderline personality disorder (BPD) in patients admitted for suicide-related behaviour	Categorise individuals with BPD into types of BPD, and evaluate characteristics of each type.	Inpatients with BPD who recently engaged in suicidal behaviours, n = 87. Mean age = 35, 100% female. Inclusion criteria: Age 18–65, no intellectual, developmental, or cognitive impairment which would impede understanding, no participants who could not understand Spanish fluently.	Clinical interview, BIS, MADRS	5% of participants were classified as an 'empty' type of BPD—lacking a stable identity or goals and reporting feelings of emptiness. The low frequency of 'empty' type of BPD may reflect the difficulty in defining and assessing emptiness, and the difficulty that people with BPD may experience in understanding the term 'emptiness'.	Clinical interview
Richman & Sokolove, 1992 [113]	The experience of aloneness, object representation, and evocative memory in borderline and neurotic patients	Test clinical observations of experience of aloneness, object representation, and evocative memory in BPD.	Outpatients with a diagnosis of BPD or neurotic disorders, n = 40 (nBPD = 20, nNeurotic = 20). Inclusion criteria: Age 18–60, no intellectual, developmental or cognitive impairment which would impede understanding, no current substance use.	Spitzer Borderline Scale, Turner Scale, WMS, HSCL-90, Rorschach Developmental Level Scale, EMT, UCLA loneliness scale (modified)	Individuals with BPD demonstrated more pervasive experiences of aloneness and lower memory quotients compared to the neurotic individuals. Memory quotient and experience of aloneness contributed 46% of the variance in predicting membership to BPD or neurotic group. Individuals with BPD experienced aloneness more frequently and more severely than neurotic individuals.	UCLA Loneliness scale
Rippetoe et al., 1986 [114]	Interactions between depression and borderline personality disorder: A pilot study	Assess overlap of symptoms between BPD and affective disorders and identify BPD symptoms associated with Axis I disorders.	Inpatients at psychiatric unit who met three or more criteria for BPD, n = 43. 54% female. Inclusion criteria: No intellectual, developmental or cognitive impairment which would impede understanding, no psychosis.	Patient chart review, DIB	Individuals with comorbid BPD and depression showed more severe chronic emptiness and boredom and more suicide attempts than individuals with BPD only.	DIB
Rogers et al., 1995 [115]	Aspects of depression associated with borderline personality disorder	Examine relationships between BPD and aspects of depression (boredom, emptiness, abandonment fears, self-condemnation, self-destructiveness, cognitive dysfunction, hopelessness, guilty, sense of failure, somatic complaints, and hopelessness).	Inpatients in public psychiatric hospital meeting criteria for depression, BPD or ASPD, n = 50 (nBPD = 16). Mean age = 27, 100% Caucasian. Inclusion criteria: No intellectual, developmental, or cognitive impairment which would impede understanding, no psychosis.	HDRS, DIB, MCMI, SDS, BDI, items from: CRS, clinical psychopharmacology research group scale, HSCL-90, IDS-SR, borderline and antisocial scales from the Personality Interview Questions	BPD groups had significantly higher emptiness and hopelessness compared to both ASPD and depression. Depression associated with BPD is phenomenologically distinct from depression or ASPD, and includes aspects of emptiness and self-condemnation.	MCMI, DIB
Sagan, 2017 [116]	The loneliness of personality disorder: a phenomenological study	Investigate and understand the experience of loneliness for people with BPD.	Participants engaged in mental health online networks with a diagnosis of BPD, n = 7, aged 25–61.	Qualitative narrative interview	Compared to samples of participants with other mental health difficulties, participants with BPD viewed loneliness as an inherent trait which is related to an inability to feel connected to the world or other people. Participants with BPD described efforts to foster connection including work or creative pursuits which provided only short-term relief from the feeling of 'un-relatedness'.	Themes from qualitative responses
Sanislow et al., 2000 [117]	Factor analysis of the DSM-III-R borderline personality disorder criteria in psychiatric inpatients	Examine factor structure of BPD in young adult inpatients.	Adult inpatients with BPD at Yale Psychiatric Institute, n = 141. Mean age = 22.4, 53% male, 89% Caucasian. Inclusion criteria: Complete inpatient data available regarding BPD.	PDE	The first of three factors was named 'disturbed relatedness', which comprised unstable relationships, identity disturbance and chronic emptiness. Disturbed relatedness reflects difficulties with relationship to self and others and may comprise the core difficulty of BPD as incomplete sense of self.	PDE
Scheel et al., 2013 [118]	Effects of shame induction in borderline personality disorder	Identify if people with BPD experience stronger and more persistent shame reactions to stimuli compared to participants with MDD and HC.	Inpatients or outpatients with BPD, participants with MDD from Rehabilitation Centre, and HC from community, n = 73 (nBPD = 25, nMDD = 25, nHC = 23). Mean age = 30.5, 100% female.	BDI, TOSCA, ZAN-BPD, developed strength of emotion questions, developed shame-inducing narrative	There was no difference in levels of boredom following shame induction exercise in BPD, MDD or HC groups.	Developed strength of emotion questions
Silk et al., 1995 [119]	Borderline personality disorder symptoms and severity of sexual abuse	Understand the relationship between severity of child sexual abuse experiences and specific BPD symptoms.	Inpatients with BPD, n = 41. Mean age = 29.1, 88% female. Inclusion criteria: No psychosis, organic disorders, no participants who could not understand English fluently.	DIB, FEI, SASSb, HDRS	Sex with a parent during childhood was predictive of feelings of hopelessness and worthlessness for both males and females with BPD. Sex with a parent was predictive of intolerance of being alone for females with BPD.	DIB
Skinstad et al., 1999 [120]	Rorschach responses in Borderline Personality Disorder with alcohol dependence	Examine differences in Rorschach responses between groups with alcohol dependence and; BPD, PDNOS or BPD and another PD.	Male inpatients in alcohol detoxification centre with BPD, PDNOS, or BPD and another PD, n = 43 (nBPD = 19, nPDNOS = 14, nBPD+OPD = 10). Inclusion criteria: No neurological disease, acute stress reaction or primary use of other substances.	Patient charts (behavioural observation, multi-disciplinary assessment), Rorschach test	Both BPD groups showed a response pattern consistent with withdrawing from social interactions and isolation.	Rorschach test
Soloff et al., 2002 [121]	Childhood abuse as a risk factor for suicidal behaviour in borderline personality disorder	Determine if childhood abuse is a risk factor for suicide in BPD, and if it is related to other risk factors for suicide in BPD.	Inpatients and outpatients of the Western Psychiatric Institute and clinic, and community members with BPD, n = 61. Mean age = 28.2, 82% female.	IPDE, DIB, SCID-I, abuse history, BHS, BIS, BGA, MMPI-PD	For people with BPD who experienced childhood sexual abuse, risk of adult suicide was increased by severity of hopelessness.	BHS
Soloff et al., 2000 [122]	Characteristics of suicide attempts of patients with major depressive episode and borderline personality disorder: A comparative study	Compare aspects of psychopathology in groups with (a) BPD, (b) MDD, and (c) BPD and MDD to determine what predicts lifetime number of suicide attempts or suicidal behaviours.	Inpatients meeting DSM criteria for MDD, BPD or both diagnoses n = 158 (nBPD = 32, nBPD+MDD = 49, nMDD = 77). Mean age = 32, 65% female, 81% Caucasian. Inclusion criteria: Age 18–83, no psychosis, no other mood disorder.	SCID I, IPDE, SIS, Lethality Scale, HAM-D, BDI, BHS, BDHI, BIS, MMPI, BGLHA, GAS	Hopelessness, lifetime number of suicide attempts, and history of aggression predicted suicide attempts for BPD group. Across groups, lethal intent in suicide attempt was predicted by hopelessness. In the BPD+MDD group an increase in hopelessness predicted increase in objective suicide planning. Increased levels of hopelessness are predictive of lifetime number of suicide attempts.	IPDE
Southward & Cheavens, 2018 [123]	Identifying core deficits in a dimensional model of Borderline Personality Disorder features: A network analysis	Examine network structure of BPD and identify core features of BPD, differences between participants high in BPD features compared to low features, and the differences in structure of BPD between gender.	Participants enrolled in eighteen studies including general population, undergraduate students and participants seeking psychological treatment, n = 4636. Mean age = 22.6, 61.1% female, 74.2% Caucasian. Inclusion criteria: Age 18+.	DERS, IIP, PAI-BOR	Network analysis of BPD features found chronic feelings of emptiness from the Identity Disturbance subscale was the most central node of the network. The node chronic emptiness had a significantly greater strength score that all other nodes except self-harm. Feelings of chronic emptiness were also found to be the most representative item of BPD from the Identity Disturbance subscale of PAI-BOR. In the high BPD features group, loneliness was a central feature of the network.	PAI-BOR
Speranza et al., 2012 [124]	Factor structure of borderline personality disorder symptomatology in adolescents	Explore factor structure of BPD DSM-IV criteria in adolescents.	Inpatients and outpatient adolescents with BPD, n = 107. Mean age = 16.6, 89% female. Inclusion criteria: No schizophrenia, chronic serious medical illnesses, no intellectual, developmental, or cognitive impairment which would impede understanding.	SIDP-IV, K-SADS-PL	Two factor solution included internally and externally oriented criteria. The internal factor included chronic feelings of emptiness, abandonment fears, identity disturbance, paranoid ideation. This factor may reflect the difficulties with experience of self during adolescence for people with BPD.	SIDP-IV
Stanley et al., 2001 [125]	Are suicide attempters who self-mutilate a unique population?	Compare suicidal behaviours for people with BPD who have a history of self-harm and no history of self-harm.	Individuals with cluster B personality disorder who had made at least one suicide attempt, n = 53. Mean age = 30, 79% female, 89% Caucasian. Inclusion criteria: No current substance use, history of head trauma, no intellectual, developmental or cognitive impairment which would impede understanding.	SIS, SADS, SIB, BGLHA, BDHI, HDRD, BHS, BPRS	BPD participants with a history of self-harm showed significantly higher scores of hopelessness and depression compared to BPD participants without self-harm history.	BHS
Stepp et al., 2009 [126]	Interpersonal and emotional experiences of social interactions in borderline personality disorder	Assess quality of social interactions and the related emotional experience for people with BPD compared to people without PD or with another PD.	Outpatients at Western Psychiatric Institute and Clinic with BPD, other PD, or no PD, n = 111 (nBPD = 42, nOtherPD = 46, nNoPD = 23). Mean age—37.5, 78.4% female, 72.1% Caucasian. Inclusion criteria: Age 21–60, no intellectual, developmental or cognitive impairment which would impede understanding, no illnesses impacting central nervous system.	SCID-I, SCID-II, Social Interaction Diary,	Participants in the BPD group experienced higher levels of emptiness compared to other PD and no PD groups. The BPD group endorsed more severe emptiness during social interactions in relation to romantic partners, family, and friends compared to other groups	SCID-II
Stiglmayr et al., 2005 [127]	Aversive tension in patients with borderline personality disorder: a computer‐based controlled field study	Evaluate if participants with BPD report higher, more frequent, more rapid or more long-lasting aversive tension compared to HC.	Inpatients and outpatients with BPD and healthy controls, n = 110 (nBPD = 63, nHC = 40). Mean age = 27.6, 100% female. Inclusion criteria: No diagnosis of schizophrenia or BP disorder, no current substance use disorder.	SCID-II, DIB-R, SCID-I, severity of aversive tension and preceding state	The events 'being alone', 'rejection', and 'failure' accounted for 39% of all events preceding aversive states for the BPD group.	Participant self-report of aversive events
Taylor & Reeves, 2007 [128]	Structure of borderline personality disorder symptoms in a nonclinical sample	Explore factor structure of BPD criteria in a nonclinical sample.	University students and general population with at least one BPD symptom, n = 82. Mean age = 18.1, 63% female, 68% Caucasian.	SIDP-IV, SCID-II	Endorsing the symptom of chronic emptiness had the highest correlation with a probable diagnosis of BPD. The first factor of analysis was named 'self-other instability' and included chronic emptiness, unstable relationships, identity disturbance, fear of abandonment, and suicidal or self-harm behaviour. This may reflect a pattern where people with BPD try to cope with feelings of emptiness and avoid abandonment by engaging in self-harm or suicidal behaviours. Chronic emptiness may result from instability in identity and relationships.	SCID-II, SIDP-IV
Taylor & Goritsas, 1994 [129]	Dimensions of Identity Diffusion	Determine the factor structure of identity diffusion, and understand the relationship between these factors and psychopathology.	Individuals responding to advertisements in local newspaper and University campus, n = 101. Mean age = 29, 64% female, 75% Caucasian. Inclusion: Age 18–65.	Identity Diffusion Interview, SCID II, PDQ-R, STAI (trait only), SCL-90-R	Core identity diffusion was related to a range of personality pathology including BPD, emptiness, and boredom.	SCID II
Thome et al., 2016 [130]	Confidence in facial emotion recognition in borderline personality disorder	Assess how people with BPD judge the intensity of emotions when presented with differing intensities of facial expressions and identify the level of confidence in the judgement.	Participants with BPD and healthy controls, n = 72 (nBPD = 36, nHC = 36). Mean age = 26.7, 100% female. Inclusion criteria: No bipolar or psychosis, substance use, pregnancy, no intellectual, developmental or cognitive impairment which would impede understanding, no psychotropic medication.	IPDE, SCID-I, BSL, BDI, SES, RSQ, Raven Test, UCLA, STAXI, ratings of intensity of emotions and level of confidence in ratings.	In the BPD group, lower confidence in rating happy faces was associated with higher levels of loneliness and higher expectations of social rejection (higher levels of rejection sensitivity).	UCLA Loneliness scale
Trull & Widiger, 1991 [131]	The relationship between borderline personality disorder criteria and dysthymia symptoms	Assess the relationship between BPD symptoms and dysthymia symptoms.	Inpatients in psychiatric hospital admitted for aggressive, psychotic or suicidal behaviour, n = 391. Mean age = 37, 42% female, 91% Caucasian. Inclusion criteria: No intellectual, developmental, or cognitive impairment which would impede understanding.	Patient charts (admission history, psychosocial history, symptom checklist).	A strong positive relationship was found between recurrent suicidal behaviour and chronic emptiness or boredom. Significant associations were found between diagnosis of dysthymia and emptiness or boredom, affective instability, suicidal behaviour, and efforts to avoid abandonment.	Presence or absence of chronic emptiness in psychiatric chart
Vardy et al., 2019 [132]	Development and validation of an experience of time alone scale for borderline personality disorder	To investigate the experience of time alone for individuals with BPD and develop a measure that reflects the experience. To then evaluate the developed measure in terms of validity and reliability.	Study 1: Participants diagnosed with BPD attending outpatient treatment, n = 12. Mean age = 36.3, 100% female. Study 2: Participants with BPD and healthy controls, n = 217 (nBPD = 112, nHC = 105). Mean age = 37.5, 88% female. Inclusion criteria: Age 18+, BPD diagnosis (BPD group).	MSI-BPD, HEI-R, AEMS, MGI-5, ETAS	Intolerance of aloneness is a key feature for individuals with BPD. Participants described feelings of helplessness and distress when alone, but also a need to escape from the demands and expectations of others. Being alone and being with others are both dysregulating.	AEMS, HEI-R, ETAS
Verardi et al., 2008 [133]	The personality profile of borderline personality disordered patients using the five-factor model of personality	Analyse the personality profile of people with BPD according to the five-factor personality model.	Outpatients referred to specialist treatment program for BPD, n = 52. Mean age = 30.4, 86.5% female.	IPDE, BFQ, BDI, BHS	Severity of hopelessness and depression did not correlate with the borderline scale of IPDE, or mediate the relationship between personality and personality disorder.	BHS
Verkes et al., 1998 [134]	Platelet serotonin, monoamine oxidase activity, and [3H] paroxetine binding related to impulsive suicide attempts and borderline personality disorder	Examine the relationship between impulsivity in borderline personality disorder and platelet indicators of central serotonergic function.	Individuals with BPD in emergency department for suicide attempt, with at least one prior additional suicide attempt, n = 144. Mean age = 35.4, 65% female. Inclusion criteria: Age 18+, no intellectual, developmental or cognitive impairment which would impede understanding, no antidepressant use, alcohol and substance dependence, no MDD or BP.	SIS, EASI-III, PDQ-R, blood samples	Chronic emptiness was positively correlated with platelet 5-HT levels. Patients with chronic emptiness, affective instability and identity disturbance comprised the largest proportion of 'grand repeaters' - 4 or more suicide attempts.	PDQ-R
Villeneuve & Lemelin, 2005 [135]	Open-label study of atypical neuroleptic quetiapine for treatment of borderline personality disorder: Impulsivity as main target	Evaluate safety and efficacy of use of quetiapine for individuals with BPD.	Outpatients with a diagnosis of BPD, n = 34. Mean age = 33.7, 73.5% female. Inclusion criteria: Aged 18–60, GAF score of less than 55, no current major depression or substance dependence, no psychosis or BD, no major medical illnesses, no women who were pregnant or of child-bearing age who were not actively taking contraceptives.	DIB-R, UKU, ESRS, BIS, BDHI, BHS, HAM-D, HARS, BPRS, TCI, SAS, GAF	Following 12 weeks of quetiapine, there was no significant reduction in severity of hopelessness.	BHS
Wedig et al, 2013 [136]	Predictors of suicide threats in patients with borderline personality disorder over 16 years of prospective follow-up	Identify predictors of suicide threats in individuals with BPD and other PDs.	Inpatients at McLean Hospital who participated in the McLean Study of Adult Development, n = 290. Mean age = 27, 80% female, 87% Caucasian. Inclusion criteria: Age 18–35, no intellectual, developmental, or cognitive impairment which would impede understanding, no BD or psychosis, fluent in English.	SCID I, DIB-R, DIPD-R, DAS, presence of suicide threat	Feeling hopeless and abandoned and engaging in behaviours of demandingness and manipulation predicted suicide threats.	DIB
Westen et al., 1992 [137]	Quality of depressive experience in borderline personality disorder and major depression: When depression is not just depression	Understand the experience of depression in a sample of individuals with BPD.	Inpatients at University of Michigan Medical Centre meeting criteria for BPD or MDD, n = 47 (nBPD = 33, nMDD = 14). 72% female. Inclusion criteria: No psychosis, no intake of pharmaceutical medicine within a two week period.	DIB-R, RDC, HAM-D, DEQ, Borderline Depression Factor (developed)	BPD groups with and without MDD can be discriminated from MDD groups by the quality of their depressive experiences with a higher rate of emptiness, loneliness, negative affect, self-concept disturbance, fears of abandonment and interpersonal difficulties. Higher severity of feelings of emptiness, dependency, and rejection sensitivity is associated with more severe depression in BPD.	DIB
Yen et al., 2009 [138]	A 5-day dialectical behaviour therapy partial hospital program for women with borderline personality disorder: predictors of outcome from a 3-month follow-up study	Identify improvement and predictors of outcome following 5-day DBT program over three month follow-up.	Individuals with BPD receiving brief partial-hospitalisation DBT, n = 47. 100% female. Inclusion criteria: Age 18–65, no intellectual, developmental or cognitive impairment which would impede understanding, no psychosis or BP, no substance use disorder.	SCID-II.BDI, BHS, DES, STAXI, BSI (GSI subscale), SIQ.	Participants endorsing chronic emptiness showed improvement in psychopathology, dissociation, and depression over the follow-up, while three participants not endorsing emptiness significantly deteriorated. Meeting criteria for chronic emptiness was not associated with depression or hopelessness scores. Emptiness may be targeted by mindfulness skills of DBT and by provision of caring, engaged, and empathetic staff.	SCID-II, BHS
Zanarini et al., 1998 [139]	The pain of being borderline: Dysphoric states specific to borderline personality disorder	Describe intensity and frequency of dysphoric states for people with BPD.	Inpatients at McLean Hospital meeting criteria for PD, n = 180 (nBPD = 146, nOtherPD = 34). Mean age = 28, ~80% Caucasian. Inclusion criteria: Age 18–35, no intellectual, developmental, or cognitive impairment which would impede understanding, no BD, no psychosis.	SCID II, DIB-R, DIPD-R, DAS	Dysphoric states were experienced at higher severity for individuals in BPD group. Emptiness as a dysphoric affect was experienced very frequently and for a large portion of time for individuals with BPD.	SCID II, DIB
Zanarini et al., 2016 [140]	Fluidity of the subsyndromal phenomenology of borderline personality disorder over 16 years of prospective follow-up	Assess rates of remission and recurrences of symptoms of BPD over 16 years.	Inpatients at McLean Hospital with BPD or other PD, n = 362 (nBPD = 290, nOtherPD = 72). Mean age = 27, 77.1% female, 87% Caucasian. Inclusion criteria: Age 18–35, no intellectual, developmental or cognitive impairment which would impede understanding, no psychosis or BP, fluent in English.	SCID-I, DIB-R, DIPD-R	Chronic hopelessness, loneliness, and emptiness all had low rates of remission and high recurrence over follow-up period. These may be a response to impaired function in relationships and work.	DIB-R, DIPD-R
Zanarini et al., 2007 [141]	The subsyndromal phenomenology of borderline personality disorder: A 10-year follow-up study	Understand time to remission for BPD symptoms over 10 years. Assess duration of symptoms over time.	Inpatients at McLean Hospital meeting criteria for BPD or another PD, n = 362 (nBPD = 290, nOtherPD = 72). Mean age = 27, 77% female, 87% Caucasian. Inclusion criteria: Age 18–35, no intellectual, developmental, or cognitive impairment which would impede understanding, no BD, and no psychosis.	SCID I, DIB-R, DIPD-R	Temperamental symptoms including emptiness and loneliness were slower to remit in BPD compared to acute symptoms. Median time to remission for chronic emptiness took the longest time (8–10 years), suggesting it may represent a more temperamental factor of BPD.	DIB

AAP—Adult Attachment Projective; AAQ–Acceptance and Action Questionnaire; ACS–Affective Control Scale; ACT–Acceptance and Commitment Therapy; ADP-IV–Assessment of DSM-IV Personality Disorders Questionnaire; ADU—Affective Dictionary Ulm; AEMS–Aloneness and Evocative Memory Scale; ASPD—Antisocial Personality Disorder; ASQ–Attachment Style Questionnaire; AUDADIS-IV—NIAAA Alcohol Use Disorder and Associated Disabilities Interview Schedule-IV; BAI–Beck Anxiety Inventory; BDHI—Buss-Durkee Hostility Inventory; BDI—Beck Depression Inventory; BDI-II—Beck Depression Inventory II; BEST—Borderline Evaluation of Severity of Time; BFQ–Big Five Questionnaire; BGA–Brown-Goodwin Lifetime History of Aggression; BGLHA—Brown-Goodwin Assessment for Lifetime History of Aggression; BHS—Beck Hopelessness Scale; BIS—Barratt Impulsiveness Scale; BIS-Brief–Barratt Impulsiveness Scale Brief; BIS–Background Information Schedule; BORRTI–Bell Object Relations and Reality Testing Inventory; BP–Bipolar Disorder; BPD—Borderline Personality Disorder; BPDSI-IV–Borderline Personality Disorder Severity Index; BPI–Borderline Personality Inventory; BPRS–Brief Psychiatric Rating Scale; BSI–Borderline Syndrome Index; BSI—Beck Suicide Ideation Scale; BSI—Brief Symptom Inventory; BSL–Borderline Symptom List; BSL-23—Borderline Symptom List-23; BSS–Beck Scale for Suicide Ideation; CASQ–Children’s Attributional Style Questionnaire; CCI–Combined Criteria Instrument; CES-D 10 –Centre for Epidemiologic Studies Short Depression Scale; CGI—Clinical Global Impression; CGI-M—Clinical Global Impression Modified; COPE–Cope inventory; CRS—Carroll Rating Scale for Depression; CSPS—Child Suicide Potential Scale; CTQ–Childhood Trauma Questionnaire; CTQ-SF—Childhood Trauma Questionnaire Short Form; DAS—Dysfunctional Attitudes Scale, DAS—Dysphoric Affect Scale; DASS-21—Depression Anxiety Stress Scales; DD—Dissociative Disorder; DEQ—Depressive Experiences Questionnaire; DERS—Difficulties in Emotional Regulation Scale; DES–Dissociative Experiences Scale; DIB—Diagnostic Interview for Borderlines; DIB-R—Diagnostic Interview for Borderlines Revised; DICA-R-A–Revised Diagnostic Interview for Children and Adolescents; DIGS–Diagnostic Interview for Genetic Studies; DIPD-R—Diagnostic Interview for DSM-III-R Personality Disorders; DIVA–Diagnostic Interview for ADHD in Adults; DSQ–Defense Style Questionnaire; DSQ–Depressive Syndrome Questionnaire; EASI-III–Emotionality Activity Sociability Impulsivity Temperament Survey III; EASQ—Extended Attributional Style Questionnaire; EMA–Ecological Momentary Awareness; EMT—Early Memories Test; EPSIS I—European Parasuicide Study Interview Schedule I; ERQ—Emotion Regulation Questionnaire; ESRS–Extrapyramidal Symptom Rating Scale; ETAS–Experience of Time Alone Scale; FAFSI—Forms and Function of Self-Injury Scale; FEI–Familial Experiences Interview; FFMQ–Five Factor Mindfulness Questionnaire; fMRI–Functional Magnetic Resonance Imaging; GAF—Global Assessment of Functioning; GAS—Global Assessment Scale; GHQ–General Health Questionnaire; HAM-D—Hamilton Rating Scale for Depression; HARS–Hamilton Anxiety Rating Scale; HC–Healthy Control; HDRS—Hamilton Depression Rating Scale; HEI-R–Hurvich Experience Inventory-Revised; HIT—Holtzman Inkblot Technique; HSC–Hopelessness Scale for Children; HSCL-90—Hopkins Symptom Checklist-90; ICS—Impulsiveness-Control Scale; IDS-SR—Inventory of Depressive Symptomatology Self Report; IIP–Inventory of Interpersonal Problems; IPDE—International Personality Disorder Examination; IPO–Inventory of Personality Organisation; ISAS–Inventory of Statements about Self Injury; IVE–Eysenck Impulsivity Venturesomeness Empathy questionnaire; K-SADS-PL—Schedule for Affective Disorders and Schizophrenia for School Aged Children (6–18 Years)–Lifetime Version; LOC–Locus of Control Scale; LPC-2 –Lifetime Parasuicide Count-2; LTE-Q—List of Threatening Experiences Questionnaire; MADRS–Montgomery-Asberg Depression Rating Scale; MAI—Multidimensional Anger Inventory; MAPI–Millon Adolescent Personality Inventory; MCMI—Millon Clinical Multiaxial Inventory; MDD—Major Depressive Disorder; MHI-5 –Mental Health Inventory 5; MINI—Mini International Neuropsychiatric Interview; MINI-Kid–Mini International Neuropsychiatric Interview for Children and Adolescents; MMPI—Minnesota Multiphasic Personality Inventory; MMPI-PD—Minnesota Multiphasic Personality Inventory Psychopathic deviate subscale; MMSE–Mini Mental State Examination; MOA—Mutality of Autonomy Scale; MOS–Mood Observation Scale; MPQ—Multidimensional Personality Questionnaire; MPQ-BF—Multidimensional Personality Questionnaire Brief Form; MSI-BPD—McLean Screening Instrument for Borderline Personality Disorder; MWT-B–Multiple-Choice Vocabulary Intelligence Test; NESARC–National Epidemiologic Survey on Alcohol and Related Conditions; OAS—Overt Aggression Scale; OMPP—Orbach and Mikulincer Mental Pain Scale; PAI-BOR–Personality Assessment Inventory–Borderline subscale; PBQ-BPD—Personality Beliefs Questionnaire BPD subscale; PCL-R–Psychopathy Checklist Revised; PD—Personality Disorder; PDE–Personality Disorder Examination; PDNOS–Personality Disorder Not Otherwise Specified; PDQ-R—Personality Diagnostic Questionnaire Revised; PHCSCS–Piers-Harris Children’s Self-Concept Scale; PHI–Parasuicide Harm Inventory; PID-5—Personality Inventory for DSM-5; PID-5-SF–Personality Inventory for DSM-5 Short Form; PIL-10—Purpose in Life-10 Items; PIL-SF–Purpose in Life Short Form; PSI—Parasuicide History Interview; PTSD—Post Traumatic Stress Disorder; RCMAS–Revised Children’s Manifest Anxiety Scale; RDC—Research Diagnostic Criteria; RFL–Reasons for Living; RRS–Risk Rescue Scale; RSE—Rosenberg Self-Esteem Scale; RSQ—Response Style Questionnaire; SADS—Schedule for Affective Disorders and Schizophrenia; SADS—Schedule for Affective Disorders and Schizophrenia; SAES–Spielberger Anger Expression Scale; SAS–Social Adjustment Scale; SASS–Social Adaptation Self-Evaluation; SASSb–Sexual Abuse Severity Scale (only need b if SASS above remains); SBQ–Suicidal Behaviours Questionnaire; SCID-CV—Structured Clinical Interview for DSM-IV Axis I Disorders-Clinician Version; SCID-I—Structured Clinical Interview for DSM-IV Axis I; SCID-II—Structured Clinical Interview for DSM-IV Axis II; SCIM–Self-Concept and Identity Measure; SCL-27 –Symptom Checklist 27; SCL-90-R—Symptom Checklist-90-Revised; SDS—Zung Self-Rating Depression Scale; SEQ—Self-Esteem Questionnaire; SES–Subjective Emptiness Scale; SFS–Social Functioning Scale; SHBCL–Rating scale of self-harm behaviours resulting from impulsivity (developed); SIB–Schedule for Interviewing Borderlines; SIDP-IV—Structured Interview for DSM-IV Personality; SIQ–Self-Injury Questionnaire; SIS—Suicide Intent Scale; SNI–Social Network Index; SOFAS–Social and Occupational Functioning Assessment Scale; SPD–Schizotypal Personality Disorder; SPIKE–Structured Psychopathological Interview and Rating of the Social Consequences of Psychological Disturbances for Epidemiology; SPSI-R—Social Problem-Solving Inventory-Revised; SRS—Suicide Risk Scale; SSI—Scale for Suicidal Ideation; STAI—State-Trait Anxiety Inventory; STAXI—State-Trait Anger Expression Inventory; STEPPS–Systems Training for Emotional Predictability and Problem Solving; SWLS–Satisfaction With Life Scale; TAAD–Triage Assessment for Addictive Disorders; TAU–Treatment as Usual; TCI—Temperament and Character Inventory; TOSCA–Test of Self Conscious Affect; TRES–Therapeutic Relationship Evaluation Scales; UCLA Loneliness Scale—University of California Los Angeles Loneliness Scale; UKU–UKU Side Effect Rating Scale; WAI-S—The Working Alliance Inventory-Short Form; WCCL–Ways of Coping Checklist; WHODAS–World Health Organisation Disability Assessment Schedule; WHOQOL-BREF–World Health Organisation Quality of Life Questionnaire; WSS—Westmead Severity Scale; WURA–Wender Utah Rating Scale; YAMI–Young Atkinson Mode Inventory YRBS—Youth Risk Behaviours Survey; YSQ–Young Schema Questionnaire; ZAN-BPD—Zanarini Rating Scale for Borderline Personality Disorder

#### Study focus

Thirty studies chosen for inclusion focused on chronic feelings of emptiness. A further 14 studies focused on chronic feelings of emptiness in addition to at least one other symptom (i.e. chronic feelings of emptiness and hopelessness, chronic feelings of emptiness and loneliness). Thirty-one studies reported on feelings of hopelessness, eight studies reported on loneliness, one study reported on loneliness and aloneness, six studies focused on aloneness, four studies focused on isolation, three studies reported on alienation, and two studies focused on boredom.

### Key findings from studies focusing on chronic emptiness

The findings from 99 included studies were categorised according to construct and key findings were extracted. The forty-four studies which focused on chronic feelings of emptiness alone or in conjunction with another key word were analysed, then key findings for similar constructs including hopelessness and loneliness were analysed separately.

#### Difficulties in defining chronic emptiness

A predominant finding of this review was the difficulty in understanding and defining the nature of chronic emptiness, and inconsistent findings regarding its relation to other symptoms of BPD. Studies investigating symptom clusters in BPD were highly variable in their results, theorising chronic emptiness was a component of: psychological process [50], affective instability [52], painful affect and defenses [63], disturbed relatedness [117], internally oriented criteria [124], and self-other instability [128]. These disparate results may be indicative of the absence of a working definition of emptiness in the field, and associated difficulties in measurement. Similarly, when investigating discriminative symptoms for a diagnosis of BPD and networks of symptoms, emptiness was often identified as an important symptom for distinguishing people with BPD from other samples [66, 103, 104, 123]. However, one study found that chronic emptiness was the least distinguishing factor of BPD [83]. The authors of this study noted that this result may be more reflective of the lack of definition of emptiness, and the difficulty in rating an internal experience that may have little behavioural manifestations, in comparison to symptoms such as unstable relationships.

Two studies discussed the difficulty of defining chronic emptiness. One study suggested that people with BPD may have difficulty defining and articulating the experiences of emptiness [112], while the other study found low correlations between chronic feelings of emptiness and other BPD symptoms, and postulated this may be due to the absence of a definition of chronic emptiness [83]. Only one recent study investigated what the features of chronic emptiness entails [111]. This study reported on feelings of emptiness transdiagnostically, and determined core features of emptiness include a sense of detachment from self and others, hollowness, aloneness, disconnection, and unfulfillment. Some studies examined the relationship of chronic feelings of emptiness as a construct to similar terms, with mixed findings. One study found no significant association between chronic feelings of emptiness and hopelessness or depression [138]. However, another study found that there were high correlations between feelings of emptiness and feelings of hopelessness, isolation, and loneliness (although these correlations did not meet multicollinearity, suggesting the construct of emptiness was still distinct) [85]. Overall, this points to a lack of cohesion in the field and a sense of confusion regarding not only the experience of emptiness, but its boundaries with related concepts.

#### Measurement of chronic emptiness within studies

In several studies, chronic emptiness was quantified by one item from a wider measure of all BPD symptoms, including structured clinical interviews. The UCLA loneliness measure was used to measure both loneliness and emptiness [113], despite some studies differentiating these concepts [85].

One scale providing some measure of emptiness is the Orbach and Mikulincer Mental Pain Scale (OMMP), which aims to measure mental pain [142]. One factor of the OMMP is labelled emptiness–measuring the loss of subjective and personal meaning due to mental pain. The emptiness factor, however, only explains 2.3% of variance in the scale and the items have not been validated as individual measures of emptiness. As such, inferring severity of chronic feelings of emptiness from the OMMP may not accurately capture the experience of chronic emptiness in BPD.

Price and colleagues [111] recently developed a transdiagnostic measure of emptiness. The resultant Subjective Emptiness Scale (SES) is a seven item self-report measure. Internal consistency of items were high across clinical samples of people with psychiatric disorders (.91-.93) and covariance analyses indicated a unidimensional construct which was able to discriminate people who experienced varying severity of emptiness. The scale included central features of emptiness as a ‘pervasive and visceral sense of detachment spanning intrapersonal, interpersonal, and existential domains of experience’ which results in ‘encompassing feelings of hollowness, absence from one’s own life, profound aloneness, disconnection from the world, and chronic unfulfillment’ [111, p. 18]. The development of this measure represents a significant contribution to the field, but as it is a transdiagnostic measure it requires validation within a BPD sample to test the symptom of chronic feelings of emptiness. Overall, the difficulties with defining and measuring chronic emptiness may partly explain the mixed findings within many reviewed studies, and points to further research aimed at elucidating the nature of chronic emptiness and the use of appropriate measures.

#### Age and gender

The prevalence of chronic feelings of emptiness was found to be higher in females with BPD compared to males [78], however a study of parent ratings of BPD in their male sons found that emptiness was reported for 97% of the male BPD group compared to 8% of the control group [74]. This study, however, did not compare genders and was reliant on parent report rather than self-report. It is not possible to provide a judgement of the effect of gender on chronic emptiness, and more study is needed in this area. One study found that chronic emptiness was more severe in older adults compared to younger adults with BPD [97]. Several factors may influence this–firstly, more ‘acute’ symptoms tend to resolve more quickly while emptiness is more chronic [141]. Perhaps once there is an absence of acute symptomology, chronic emptiness is more noticeable or more severe. Secondly, older adults in this study had poorer social function, which possibly results from a sense of disconnection from others and a feeling of emptiness.

#### Detachment from self and others

The limited number of studies on emptiness as a disconnection or deficiency in relating to self and others was surprising given the theoretical import placed on this relationship. The strongest support for this model was found by Price and colleagues [111] who proposed that transdiagnostic emptiness was a sense of detachment from interpersonal, intrapersonal and existential spheres. In terms of detachment from self, three studies linked chronic emptiness to identity disturbance, reflecting a detachment from sense of self. A qualitative study which asked for the life stories of people with BPD found an emergent theme of chronic feelings of emptiness relating to disturbances in self-identity [101]. This theme included two subthemes–distorted self-image and lack of identity–resulting in chronic emptiness. One study explored identity diffusion within personality disorder presentations, and found it was associated with feelings of chronic emptiness [129], while another study typified a subtype of people with BPD as the ‘empty’ type characterised by deficits in identity [112]. These studies suggest that chronic feelings of emptiness are the expression of an underlying diffuse identity, reflecting theoretical claims [11]. In relation to detachment from others, one study reported that social dysfunction was associated with feelings of emptiness [37]. Other studies noted that chronic emptiness occurred most often when individuals with BPD were alone [96] or during interactions with others in close social relationships [126].

#### Course of chronic emptiness

Five studies presented results relating to the course of chronic feelings of emptiness in BPD. Zanarini and colleagues [139] found that feelings of chronic emptiness were experienced frequently and severely for people with BPD. They also found that when investigating symptoms of BPD over ten years follow-up, feelings of chronic emptiness took the longest time to remit at an average of 8–10 years compared to more acute symptoms [141]. In a similar study, authors further found that over 16 years, chronic emptiness had relatively poor remission rates compared to other symptoms, and high recurrence rates [140]. These studies suggest that feelings of emptiness are difficult to alleviate due to being a ‘temperamental’ symptom enduring over time rather than an acute symptom. Similarly, another longitudinal study aiming to identify the core clinical features of BPD found that after one year of treatment feelings of emptiness were chronic compared to more acute symptoms, suggesting that chronic feelings of emptiness may represent a core underlying factor in BPD or is not targeted by current treatment [95]. A further study found that in a cohort of people with BPD categorised into a younger age group (18–25) and older age group (45–68), older adults were more likely to report chronic feelings of emptiness [97]. The authors hypothesised that chronic emptiness may be more difficult to change as people age compared to symptoms like mood dysregulation. Overall, the studies focusing on the course of chronic feelings of emptiness reported it as slow to change over time, hypothesising it is a core problem for people with BPD. However, another factor in the chronicity of emptiness could be that it is not targeted in most treatments, and as such it remains untreated for a long period of time.

#### Chronic emptiness, impulsivity, self-harm and suicide

Ten studies investigated behaviours which followed chronic feelings of emptiness. Both qualitative and quantitative studies supported chronic feelings of emptiness preceding impulsive behaviours. A qualitative study reported that women with BPD attempt to fill the ‘void’ they experience by acting impulsively [101], while a longitudinal study found that impulsivity and self-harm mediated the relationship between chronic emptiness and days out of work over time, suggesting that chronic emptiness may underlie and result in behavioural symptoms of impulsivity including self-harm [38]. A study in a sample of college students found that 67% of participants reported feelings of emptiness prior to engaging in self-harm behaviours [85]. Another study supported these findings with a different college sample, reporting that chronic feelings of emptiness and identity disturbance were associated with a history of self-harm behaviour, and may be the motivation for engaging in these maladaptive behaviours [39]. Overall, these studies may suggest that the void of emptiness is distressing and a common way to tolerate this distress is to engage in self-harm or impulsive behaviour.

Studies also reported a link between chronic feelings of emptiness and suicidal behaviours. One study hypothesised suicidal behaviours and suicide attempts are engaged in by people with BPD to relieve the tension of feeling empty inside [50]. Supporting this hypothesis, studies have found a strong relationship between chronic emptiness and both suicidal ideation and behaviour [85, 131]. In one study people with BPD who experience chronic emptiness, mood dysregulation and identity disturbance made up the largest proportion of people who had made more than three suicide attempts [134]. Another study found that the presence of chronic emptiness increased the odds of suicide attempts [75]. It is possible that when self-harm and impulsive behaviours no longer relieve the distress of emptiness, suicidal ideation and behaviours arise.

#### Chronic emptiness as linked to depressive experiences

Seven studies investigated the relationship between chronic emptiness and depressive experiences. One study reported a moderate correlation between feelings of emptiness and depression [85], while another found that individuals endorsing chronic emptiness had significantly more severe depression than those who did not experience chronic emptiness [83]. Chronic emptiness was experienced frequently as a dysphoric affect for individuals with BPD [139], and was significantly associated with a diagnosis of dysthymia [131]. Individuals with diagnoses of BPD and MDD had higher rates of chronic emptiness and suicide attempts than people who met diagnosis for BPD only [114]. Two studies viewed depression in BPD as qualitatively different to that of MDD [115, 137]. Borderline depression was characterised by chronic emptiness and self-condemnation [115]. Emptiness, rejection sensitivity, and dependency were positively associated with more severe depression in BPD which was also related to disturbances of self-concept [137].

#### Impact of chronic emptiness on social and vocational function

Several studies discussed the impact of chronic feelings of emptiness on vocational and social functioning for people with BPD. One study hypothesised that chronic feelings of emptiness was an understandable response to a life of relational difficulties and impaired work function [140]. A study by Ellison and colleagues [37] found that people presenting for psychiatric treatment who endorsed the single chronic feelings of emptiness symptom had the poorest psychosocial outcomes–the highest number of days out of work and lowest social functioning–compared to groups with any other individual symptom of BPD. Groups with both chronic emptiness and impulsivity had missed more work in the last five years, and groups with chronic emptiness and anger had poorer social functioning compared to people presenting to care with no BPD symptoms. This supported results from a previous study which found that compared to both other personality disorder presentations and people with no personality disorder, people with BPD reported higher levels of chronic emptiness during social interactions with close relationships [126]. A recent study further found that chronic feelings of emptiness predicted days out of work or normal activities over a one year follow-up, suggesting that chronic emptiness may account for psychosocial dysfunction over time [38]. Interestingly, another study found that after investigating BPD symptoms within a community sample over three times points within 18 months, chronic feelings of emptiness were associated with less stressful life events in the preceding six months compared to more acute symptoms [110]. This is perhaps a reflection of impaired social relationships and subsequent social isolation, leading to minimal stressful interpersonal events.

#### Treatment for chronic emptiness

Three studies discussed psychological treatment of chronic feelings of emptiness. A range of therapeutic modalities were used, including Supervised Team Management plus Sequential Brief Adlerian Psychodynamic Psychotherapy [47], Systems Training for Emotional Predictability and Problem Solving (STEPPS) [56], and Dialectical Behaviour Therapy (DBT) [138]. Each of the studies found that following treatment chronic feelings of emptiness significantly decreased in BPD samples. The follow-up period of these studies ranged from three months to two years. Authors speculated that chronic emptiness may be alleviated due to an increase in mentalisation skills, decrease in idealising and devaluing patterns within relationships, and an increased capacity to tolerate ambiguity and ambivalence [47]. In the STEPPS study, identity disturbance and mood instability also decreased alongside chronic emptiness [56]. Within the DBT treatment, participants experiencing chronic emptiness at baseline (94% of the sample) improved over the three months of treatment, while participants who did not endorse chronic emptiness (6%) demonstrated statistically significant deterioration of depressive symptoms, dissociative symptoms, and general mental health [138]. Authors postulated there may be two factors influencing the change in chronic emptiness. Firstly, they speculated that the core skill of mindfulness in DBT targets feelings of chronic emptiness. Secondly, they noted the model within which DBT was practiced ‘offered a validating community to women’ [138, p. 9] with high levels of engagement between participants and practitioners, which may have increased feelings of connection with others and self. It is important to note that there is as yet no causal empirical evidence that supports these hypotheses.

### Similar constructs

#### Hopelessness

Thirty-six studies reported on hopelessness or a combination of hopelessness and another keyword. Hopelessness was typically defined as a disconnection from meaning and disconnection from life [90, 92]. Eleven studies discussed the role of hopelessness in self-harm and suicidality. Overall, severity of hopelessness was associated with suicidal behaviours [68, 107, 121, 136] and in some studies predicted suicide attempts for people with BPD [122]. Several studies focused on feelings of hopelessness as a disconnection from or lack of meaning in life. Low meaning in life was associated with more suicidal ideation and attempts, and hopelessness was also positively associated with suicidal behaviours [68, 107, 121, 122, 136]. Low meaning in life predicted hopelessness [72], and meaning in life also moderated the relationship between previous suicide attempts and hopelessness [92]. One study found that hopelessness mediated the relationship between BPD and suicide attempts [64]. For people who had attempted suicide, severity of hopelessness was higher for those who met diagnosis for BPD [53]. Within a BPD sample, individuals with a history of self-harm expressed higher severity of hopelessness compared to those without self-harm history [125].

Several studies reported on the link between feelings of hopelessness and depressive experiences. Depression was found to predict hopelessness for people with BPD, and was mediated by a sense of meaning in life [90]. Low meaning in life was correlated with feelings of both hopelessness and depression [91] and predicted both depression and hopelessness [72]. One study found people with comorbid BPD and MDD had more severe hopelessness compared to people with MDD only [46], and people with BPD had higher ratings of depression and hopelessness than people with depressive disorders [69]. However, one study found that hopelessness was unable to distinguish adolescents with and without BPD, suggesting it is not a unique experience of BPD [109]. An additional study found there were no differences in hopelessness and depression between people with BPD and people with MDD [79]. Multiple studies reported on the change in hopelessness throughout treatment for BPD. Severity of hopelessness decreased for people with BPD following DBT treatment–including both intense and adapted DBT programs [70, 80, 86, 94, 108], although one trial found DBT was not superior to Collaborative Assessment and Management of Suicidality treatment [48]. Severity of hopelessness also decreased following Acceptance and Commitment Therapy group treatment [98] and cognitive therapy [60, 67]. One study found that severity of hopelessness did not decrease following treatment with antipsychotic medication for three months [135].

#### Loneliness

Eighteen studies discussed loneliness or a combination of loneliness and another keyword. Loneliness has been conceptualised as a ‘feeling of being alone’ [89, p. 1], which is a central feature within the network of BPD symptoms [123]. One study reported that people with BPD perceive loneliness as an inherent trait, not a state, which reflects a feeling of disconnection with the world and can only be temporarily alleviated [116]. Among personality disorders, BPD had the strongest association with loneliness [77], and adolescents who self-harm reported higher rates of loneliness compared to those who did not self-harm [73]. One study reported that loneliness, in addition to chronic emptiness, was a core factor of depression for people with BPD [137], while two other studies clustered chronic emptiness, loneliness and boredom as a discriminating factor of BPD [103, 104]. Loneliness was found to have high recurrence and low remission rates over both 10- and 16-years follow-up [140, 141]. People with BPD demonstrated higher dysregulation compared to healthy controls following presentation of attachment pictures which may induce loneliness, suggesting an intolerance of loneliness [54]. Similarly, people with BPD demonstrated a higher intolerance to loneliness compared to people with dissociative or conversion disorders [105]. One study reported that loneliness in BPD was related to poor social and relational function, but after controlling for these deficits loneliness was still high for people with BPD, suggesting there are multiple factors which contribute to feeling lonely [89]. Feelings of loneliness may also be associated with deficits in facial emotion recognition and behavioural mimicry. Lower confidence in rating facial emotions has been associated with both higher levels of loneliness and higher levels of rejection sensitivity [130]. For people with BPD with the highest scores of loneliness, behavioural mimicry–an important factor in fostering connection between people–was the lowest, suggesting the capacity or desire to connect with others may be impaired when people with BPD feel lonely [76].

#### Intolerance of aloneness

Intolerance of aloneness broadly relates to the intolerable distress of being alone with one’s own thoughts and feelings and an associated incapacity for solitude [132]. Overall findings indicate that people with BPD experience the feeling of aloneness more frequently and severely compared individuals with neurotic disorders [113] and have an intolerance to being alone [61, 105]. A recent study developed a measure for the experience of being alone for individuals with BPD and they report the intolerance of this experience as a salient feature of the disorder [132]. Being alone accounted for 39% of aversive emotions [127] and triggered all BPD symptoms except self-harm [96]. Over ten years intolerance of aloneness was the slowest interpersonal symptom of BPD to remit and still declined less than other features of BPD [65]. Interestingly, an article found that both intolerance of being alone and intolerance of relating to others were salient features of the experience for people with BPD [132].

#### Alienation and boredom

Three studies reported on feelings of alienation. Alienation was found to be a discriminating feature of BPD [51] and was a risk factor for development of BPD [59]. It was also associated with disturbed identity [87]. Five studies reported on feelings of boredom or boredom in conjunction with chronic emptiness. Most of these studies were published when the symptom of chronic emptiness or boredom remained in the DSM. Boredom was found to be related to core identity diffusion [129], and suicidal behaviour [131]. Boredom was also associated with feelings of depression [81, 114], however was not associated with feelings of shame [118].

## Discussion

This review sought to examine empirical literature and provide a detailed understanding of the symptom of chronic feelings of emptiness in BPD. It also aimed to identify similar constructs to chronic feelings of emptiness such as hopelessness, and provide clarification around the relationship between these experiences. A broad focus was used in this review–articles needed to be peer-reviewed, contain novel empirical data, and needed to have a focus on BPD or BPD symptoms. However, all articles that mentioned emptiness or a similar construct in their abstract and results or discussion were included, even if the main focus of the study was not on these experiences. This allowed an in-depth analysis within a field where chronic feelings of emptiness is often discussed tangentially and is not a common focus of articles. However, this also resulted in a wide array of study methodology and quality, and findings should be interpreted with caution until further research is conducted.

Overall, 99 articles met the inclusion criteria and quality assessment, and key findings were presented. The review identified a number of gaps within the literature, particularly relating to defining and measuring chronic feelings of emptiness. As such, findings extrapolated from this data should be interpreted with caution, as there are significant limitations with measurement within the field. Nevertheless, the included studies provide a good foundation of knowledge regarding chronic feelings of emptiness.

### The difficulty in defining and delineating chronic feelings of emptiness

The available research on chronic feelings of emptiness demonstrated a difficulty in understanding the nature of chronic emptiness, defining the experience, and determining its importance to a BPD conceptualisation or diagnosis. Despite the inclusion of 44 studies discussing chronic feelings of emptiness, only one recent study investigated what chronic emptiness is and how it is experienced, although this was not exclusive to individuals with BPD but included all psychiatric diagnoses [111]. It is clear from included studies that it remains difficult to define and measure an absence of experience, and perhaps this has resulted in the reliance on single-item measures that may not adequately capture the true experience of chronic emptiness. Factor analyses differentially placed chronic emptiness with most other symptoms of BPD, perhaps a further indication of the absence of a definition of chronic emptiness. There were minimal personal accounts of people with BPD across the studies. Only three qualitative studies focused on individual experiences, with most other studies utilising prescribed questions which are often developed by clinicians or researchers and may not accurately reflect the experience of individuals with BPD. The lack of understanding about the nature of chronic emptiness may also contribute to the mixed findings of chronic feelings of emptiness within the broader conceptualisation of BPD.

### A conceptualisation of the cause and effect of chronic feelings of emptiness within BPD

Despite difficulties defining and delineating chronic emptiness, this review is able to provide a synthesis on the current understanding of chronic emptiness in the theoretical and empirical literature. Across differing theoretical frameworks, a common theme in the conceptualisation of chronic emptiness is that it results from a disconnection from the self and from other people. This is described differentially in terms of unstable object relations [8, 15, 16, 143, 144], an inability to develop soothing and holding introjects [22], a false self [23], a lack of personal identity [26, 27, 29], insecure attachments [30], invalidation and confusion about internal experiences [31] and deficits in mentalisation [32]. These theories hypothesised the cause of emptiness is inconsistent responses from caregivers resulting in difficulties in knowing oneself and others. Empirical literature that focused on emptiness as a sense of detachment from self and others was not detailed enough to be conclusive, but provided some empirical indications that support these theories. In particular, Price and colleagues [111] found a unidimensional construct of emptiness that was defined as a sense of detachment both from self and others, hollowness, aloneness, disconnection, and unfulfillment [111]. Qualitative narratives have begun to demonstrate in small samples that people with BPD may also associate feelings of chronic emptiness with identity disturbance [101]. Further, treatment that focuses on establishing a more coherent sense of identity and empathic responding to others (e.g. mindfulness [138], mentalisation [47]) also appears to decrease the severity of chronic emptiness, suggesting a possible link between chronic emptiness and disconnection from self and others.

The research was more conclusive on the effects of emptiness for people with BPD. Chronic emptiness was linked to several aversive outcomes including vocational and social function [37, 38], impulsivity, self-harm [85] and suicidal behaviours [75]. A review of the relationship between emptiness and suicidal behaviour found that feelings of emptiness was among the most frequent affect experienced before suicide attempt and after non-fatal suicide attempts [36]. It is possible that deficits in connecting with oneself and others leads to an intolerable sense of emptiness, which is avoided or alleviated by engaging in self-destructive behaviours. Likely, both the feelings of detachment from self and other people and the resultant behaviours impair both social and vocational functioning.

The experience of chronic emptiness has been conceptualised as a component of depression in BPD [40, 115, 145]. Depression has never been a criterion for meeting a diagnosis of BPD [2, 6], but there is high occurrence of both reported depressive experiences and diagnosable depressive disorders including major depressive disorder (MDD) in BPD [146, 147]. There are also indications, however, that there exists a ‘borderline depression’ which is qualitatively different to the experiences of affective disorders [9]. Current theoretical models purport that the experience of depression in BPD is intrinsically linked to an insecure and negative self-identity, which is exacerbated by dysregulation of emotion, anger, anxiety, and importantly–emptiness [9, 40]. Borderline depression is centred on these experiences of loneliness, anger, impaired self-concept and relationships rather than the characteristic feeling of guilt in MDD [40, 137, 146, 148]. Specifically, it is suggested a discriminating factor between borderline depression and unipolar depression is the experience of emptiness [145]. Borderline depression is hypothesised as a ‘feeling of isolation and angry demandingness rather than true depression’ [9, p. 36] and represents a more dependent-anaclitic form of depression [20]. This is considered distinct from other depressive disorders, and reflects an experience where a common characteristic is feelings of chronic emptiness.

The proposition that chronic emptiness is a component of ‘borderline depression’ still needs to be clarified in future research, but at the very least there is a positive association between chronic feelings of emptiness in BPD and severe depression [115, 137]. Two studies which investigated the experience of depression in BPD found that a ‘borderline’ depression was associated with poor self-concept and a sense of ‘void’ or ‘inner badness’ [115, 137]. These feelings of chronic emptiness and perhaps the experience of borderline depression may then result in impulsive behaviours including self-harm or suicidal behaviours to reduce the feeling of emptiness or depression [38, 39, 85, 101]. The literature in this area remains inconclusive, with recent research with participants with severe and recurrent depression indicating feelings of chronic emptiness are also an important component of their experiences [149].

Research on the cause and effects of chronic emptiness highlights the importance of increasing knowledge of this symptom. Specifically Brickman and colleagues [39] suggests individuals who experience substantial feelings of emptiness should be identified and targeted for interventions, as they may be more likely to engage in maladaptive behaviours and may have a poorer functional prognosis.

### A difference in connection–separating chronic emptiness from related constructs of hopelessness, loneliness and intolerance of aloneness

There have been limited efforts to distinguish chronic feelings of emptiness from similar or related constructs. One study investigated the relationship between feelings of chronic emptiness and hopelessness, isolation, loneliness, uselessness, worthlessness, and grief before and after self-harm incidents with university students [85]. It found high correlations between feelings of chronic emptiness and feelings of hopelessness, loneliness and isolation. The authors proposed that these four states all represent a low positive affect and low rates of arousal. Other studies included chronic emptiness, loneliness and hopelessness together as temperamental affective experiences of BPD [139–141], considering them highly related symptoms of BPD.

Based on the reviewed literature, it seems that chronic feelings of emptiness may be distinguishable from similar constructs. We hypothesise that chronic feelings of emptiness is a sense of disconnection from both self and others, hopelessness is a sense of disconnection to meaning or life, loneliness is a sense of disconnection from the world and a feeling of being alone and intolerance of aloneness is the incapacity to be alone. All have a similar basis in a sense of disconnection or detachment but represent different types of disconnect. This hypothesis of emptiness as a sense of detachment and disconnection from self and others echoes that of Price and colleagues [111].

Studies which discussed feelings of hopelessness often viewed it as a disconnection from or lack of meaning in life [72, 90–92]. Less meaning in life was associated with more suicidal behaviours. Interestingly, meaning in life–a sense of purpose to life–has been shown as a factor in decreased suicidal ideation [150] and gratefulness towards life has been shown as a buffer between suicidal ideation and hopelessness [151]. Perhaps a sense of hopelessness may reflect low meaning in life and a disconnection from life.

Studies focusing on loneliness in BPD discussed it as a sense of disconnection from others that people with BPD perceive as a sense of disconnection with the world [116]. Feelings of loneliness were associated with deficits in facial emotion recognition [130] and behavioural mimicry [76]–suggesting impairments in fostering connection with other people. Loneliness may both arise from a sense of social disconnection and perpetuate deficits in social interactions. Similarly, people with BPD demonstrated an intolerance to being alone and feelings of aloneness, but also experienced being in the company of other people as dysregulating [132].

While we hypothesise that chronic emptiness, hopelessness and loneliness may be distinguishable from one another, this is based on limited data which has not explicitly investigated these differences. While Klonsky’s [85] research began the process of demarcating these experiences, further research is needed to further investigate the differences in constructs and to test the hypothesis.

### Treating the chronically empty: Hypothesising a possible treatment focus

Chronic feelings of emptiness seems to be an affective symptom of BPD that is temperamental–meaning it takes significantly longer to remit compared to more acute symptoms [139, 141]. This may be due to the nature of chronic emptiness itself, or it may be that most current treatments do not focus specifically on alleviating the symptom.

A limited number of studies discussed treatment for chronic feelings of emptiness. Those that did hypothesised that a reduction in chronic feelings of emptiness was related to an increase in mindfulness skills, mentalisation skills, and a decrease in patterns of idealisation and devaluation [47, 138]. Yen and colleagues [138] also considered the impact of validation from clinicians in fostering a sense of community and belonging to self and others. It may be that developing mindfulness skills in DBT within a supportive and safe environment fosters a sense of identity and purpose, and similarly mentalisation-based and transference-focused therapies focus on making sense of the internal world of individuals [152], their self-representations [153], and their connections to others. We hypothesise that work on self-integration including strengthening an understanding of autobiographical history, personal preferences, and sense of self as a unique personality which is allowed to just ‘be’ may have a flow-on effect and reduce the severity of chronic emptiness. Further, a focus on increasing holding others in mind in addition to basic behavioural strategies may assist in developing social connection. This speculation of the possible treatment for chronic emptiness remains a preliminary hypothesis until research can be conducted testing this specific model.

### Study design and methodological limitations

Findings within this review are dependent upon our interpretation of available data. It is important to note that articles included in the review had a wide variance in both scope and quality. In considering the limitations of the field, this systematic review is also limited by the nature of studies reporting of chronic feelings of emptiness; in that findings regarding chronic emptiness were often presented tangentially to other main findings, and as such were often not interpreted at an in-depth level within studies.

Study quality within this area of research is also limited. Few studies stated their sampling procedure or justified their sample size, and reasons why eligible participants chose not to participate were rarely stated. While this is an important area of research, our findings should be interpreted with some caution due to the differences in quality of the included studies. Most studies included in the review presented cross-sectional data (n = 73). Although cross-sectional data is an efficient way to collect data at one time point, it does not allow an analysis of change over time or causal relationships, weakening the conclusions of these articles. However, the findings from longitudinal studies (n = 23) within the included articles were generally consistent with findings from cross-sectional findings.

Despite the importance of being able to identify individuals who experience significant feelings of chronic emptiness, there has historically been a lack of comprehensive methods to measure emptiness. This may reflect the difficulty in defining or measuring an absence of experience which has been described as a sense of ‘nothing’ [9, 112]. Within included studies, there was a higher proportion of studies utilising measures which were specific to a BPD sample (n = 49) or both specific measures and more general measures (n = 10), compared to general measures only (n = 39). This may have allowed for investigation into features and experiences that are specific to BPD, while also allowing an understanding of difficulties with chronic emptiness or a related construct that are not unique to BPD. However, a significant weakness of the included studies is that the majority of articles employed a single-item measure to quantify presence or severity of chronic feelings of emptiness or a related experience. Emptiness has typically been measured using one individual item from semi-structured interviews or diagnostic tools [154–156]. This may not adequately capture the nature and severity of chronic emptiness and restricts generalisability of findings. Themes arising from the data in this review should be interpreted cautiously due to the limitation of single-item measurements. The recent development of the transdiagnostic Subjective Experience of Emptiness scale [111] provides a good future direction for further studies which require a more thorough and in-depth understanding of feelings of chronic emptiness.

### Implications for future research

The findings of this review support several areas of further research. First, there is a need to better understand the nature of chronic emptiness for people with BPD. Qualitative studies are needed to provide an in-depth account of the personal experience of chronic feelings of emptiness to support the development of better ways to measure or quantify chronic emptiness. Second, research in this area could expand on the recent work of Price and colleagues [111] to validate their transdiagnostic measure of emptiness in a BPD sample or add an extension to this measure that is specific to people with BPD. It may be of use to explore transdiagnostic research into emptiness for other presentations, such as chronic depression [149], eating disorders and substance use to further inform our understanding of and interventions for emptiness. Third, once there is a more thorough understanding of chronic feelings of emptiness and a way to quantify its presence and severity, we may be able to test intervention models targeting chronic emptiness.

Despite the inclusion of chronic feelings of emptiness as a diagnostic marker for BPD, it has not been subjected to the same level of interrogation as other symptoms of BPD. This review provided a detailed analysis on literature regarding the construct of chronic feelings of emptiness. Results demonstrated that while there remains many gaps in our knowledge about chronic emptiness, it is clear that as a whole studies point to it as a signal symptom to consider in conceptualisation and treatment of BPD. Further studies are needed to provide a deeper understanding of chronic emptiness and its clinical significance in order to develop effective interventions.

## Supporting information

S1 Table Results of quality check using MMAT observational descriptive and qualitative tool for included studies. (DOCX)

S2 Table PRISMA 2009 checklist. (DOC)
