# Supplementary material for: Measuring the shadows: A systematic review of chronic emptiness in borderline personality disorder
Source: PLoS One. 2020 Jul 1;15(7):e0233970. doi: 10.1371/journal.pone.0233970 (PMC7329066; doi:10.1371/journal.pone.0233970)
Supplement: S1 Table — (DOCX) [file pone.0233970.s001.docx]

**S1 Table. Results of Quality Check Using MMAT Observational Descriptive and Qualitative Tool for Included Studies**

| **Authors, year** | **Screening questions** | | | **4.1 Is the sampling strategy relevant to address the quantitative research question?** | | | | **4.2 Is the sample representative of the population under study?** | | **4.3 Are measurements appropriate (clear origin, validity known, standard instrument)?** | | | | | | **4.4 Is there an acceptable response rate (>60%)?** | **Quality of study (range 0-8)** |
| --- | --- | --- | --- | --- | --- | --- | --- | --- | --- | --- | --- | --- | --- | --- | --- | --- | --- |
|  | **Are there clear research questions/ objectives?** | **Do the collected data address the research question?** | | **Is the source of sample relevant to the population under study?** | | **Is there a standard procedure for sampling/sample size is justified?** | | **Are inclusion and exclusion criteria explained?** | **Are reasons why eligible individuals chose not to participate explained?** | **Are the variables clearly defined and accurately measured?** | | **Are measurements justified and appropriate for answering the research question?** | | **Do the measurements reflect what they are supposed to measure?** | |  |  |
| Abela et al., 2003 | Y | Y | | Y | | N | | N | N | Y | | Y | | Y | | Y | 5 |
| Amianto et al., 2011 | Y | Y | | Y | | N | | Y | Y | Y | | Y | | Y | | Y | 7 |
| Andreasson et al., 2016 | Y | Y | | Y | | Y | | Y | Y | Y | | Y | | Y | | Y | 8 |
| Bach & Sellbom, 2016 | Y | Y | | Y | | N | | N | N | Y | | Y | | Y | | Y | 5 |
| Becker et al., 2006 | Y | Y | | Y | | N | | Y | N | Y | | Y | | Y | | Y | 6 |
| Bell et al., 1988 | Y | Y | | Y | | N | | Y | N | Y | | Y | | Y | | Y | 6 |
| Benazzi, 2006 | Y | Y | | Y | | N | | N | N | Y | | Y | | Y | | Y | 5 |
| Berk et al., 2007 | Y | Y | | Y | | N | | Y | N | Y | | Y | | Y | | Y | 6 |
| Bernheim et al., 2018 | Y | Y | | Y | | N | | Y | N | Y | | Y | | Y | | Y | 6 |
| Bhar et al., 2008 | Y | Y | | Y | | N | | Y | N | Y | | Y | | Y | | Y | 6 |
| Black et al., 2018 | Y | Y | | Y | | N | | Y | N | Y | | Y | | Y | | Y | 6 |
| Bohus et al., 2007 | Y | Y | | Y | | N | | N | N | Y | | Y | | Y | | Y | 5 |
| Bohus et al., 2001 | Y | Y | | Y | | N | | N | N | Y | | Y | | Y | | Y | 5 |
| Bornovalova et al., 2006 | Y | Y | | Y | | N | | Y | N | Y | | Y | | Y | | Y | 6 |
| Brickman et al., 2014 | Y | Y | | Y | | N | | Y | N | Y | | Y | | Y | | Y | 6 |
| Brown et al., 2004 | Y | Y | | Y | | N | | Y | Y | Y | | Y | | Y | | Y | 7 |
| Buchheim et al., 2008 | Y | Y | | Y | | N | | Y | N | Y | | Y | | Y | | Y | 6 |
| Chabrol et al., 2001 | Y | Y | | N | | N | | N | N | Y | | Y | | Y | | Y | 4 |
| Chabrol et al., 2002 | Y | Y | | Y | | N | | N | N | Y | | Y | | Y | | N | 4 |
| Chapman et al., 2005 | Y | Y | | N | | N | | Y | N | Y | | Y | | Y | | Y | 5 |
| Choi-Kain et al., 2010 | Y | Y | | Y | | N | | Y | N | Y | | Y | | Y | | Y | 6 |
| Conte et al., 1980 | Y | Y | | Y | | N | | N | N | Y | | Y | | N | | Y | 4 |
| Cottraux et al., 2009 | Y | Y | | Y | | N | | Y | N | Y | | Y | | Y | | N | 5 |
| Ellison et al., 2016 | Y | Y | | Y | | N | | Y | N | Y | | Y | | Y | | Y | 6 |
| Espinosa et al., 2009 | Y | Y | | Y | | N | | N | N | Y | | Y | | Y | | Y | 5 |
| Fertuck et al., 2016 | Y | Y | | Y | | N | | Y | N | Y | | Y | | Y | | Y | 6 |
| Flynn et al., 2017 | Y | Y | | Y | | N | | Y | N | Y | | Y | | Y | | Y | 6 |
| Fritsch et al., 2000 | Y | Y | | N | | N | | N | N | Y | | Y | | Y | | Y | 4 |
| Garcia-Alandete et al., 2014 | Y | Y | | Y | | N | | Y | N | Y | | Y | | Y | | Y | 6 |
| Glenn & Klonsky, 2013 | Y | Y | | N | | N | | Y | Y | Y | | Y | | Y | | Y | 6 |
| Goodman et al., 2013 | Y | Y | | Y | | N | | N | N | Y | | Y | | Y | | N | 4 |
| Harford et al., 2018 | Y | Y | | Y | | N | | N | N | Y | | Y | | Y | | Y | 5 |
| Hauschild et al., 2018 | Y | Y | | Y | | N | | Y | N | Y | | Y | | Y | | Y | 6 |
| Hengartner et al., 2014 | Y | Y | | N | | N | | Y | Y | Y | | Y | | Y | | Y | 6 |
| Hoertel et al., 2014 | Y | Y | | Y | | Y | | Y | N | Y | | Y | | Y | | Y | 7 |
| Horesh et al., 2003 | Y | Y | | Y | | N | | Y | N | Y | | Y | | Y | | Y | 6 |
| Hulbert & Thomas, 2007 | Y | Y | | Y | | N | | Y | N | Y | | Y | | Y | | Y | 6 |
| James et al., 1995 | Y | Y | | Y | | N | | Y | N | Y | | Y | | Y | | Y | 6 |
| Javaras et al., 2017 | Y | Y | | Y | | N | | Y | N | Y | | Y | | Y | | Y | 6 |
| Johansen et al., 2004 | Y | Y | | Y | | N | | N | N | Y | | Y | | Y | | Y | 5 |
| Kerr et al., 2018 | Y | Y | | N | | N | | N | N | Y | | Y | | Y | | Y | 4 |
| Klonsky, 2008 | Y | Y | | Y | | N | | Y | N | Y | | Y | | Y | | Y | 6 |
| Koons et al., 2001 | Y | Y | | Y | | N | | Y | Y | Y | | Y | | Y | | N | 6 |
| Korner et al., 2008 | N | N | | - | | - | | - | - | - | | - | | - | | - | 0 |
| Lenzenweger et al., 2012 | Y | Y | | Y | | N | | Y | N | Y | | Y | | Y | | Y | 6 |
| Leppänen et al., 2016 | Y | Y | | Y | | N | | Y | N | Y | | Y | | Y | | Y | 6 |
| Liebke et al., 2017 | Y | Y | | Y | | N | | Y | N | Y | | Y | | Y | | Y | 6 |
| Marco et al., 2014 | Y | Y | | Y | | N | | Y | N | Y | | Y | | Y | | Y | 6 |
| Marco et al., 2015 | Y | Y | | Y | | Y | | Y | N | Y | | Y | | Y | | Y | 7 |
| Marco et al., 2017 | Y | Y | | Y | | N | | Y | N | Y | | Y | | Y | | Y | 6 |
| McGlashan, 1987 | Y | Y | | Y | | N | | Y | N | Y | | Y | | Y | | Y | 6 |
| McQuillan et al., 2005 | Y | Y | | Y | | N | | Y | N | Y | | Y | | Y | | Y | 5 |
| Meares et al., 2011 | Y | Y | | Y | | N | | Y | N | Y | | N | | N | | Y | 4 |
| Miller et al., 2018 | Y | Y | | Y | | N | | N | N | Y | | Y | | Y | | Y | 5 |
| Miskewicz et al., 2015 | Y | Y | | Y | | N | | Y | N | Y | | Y | | Y | | Y | 6 |
| Morgan et al., 2013 | Y | Y | | Y | | N | | Y | N | Y | | Y | | Y | | Y | 6 |
| Morton et al., 2012 | Y | Y | | Y | | N | | Y | N | Y | | Y | | Y | | Y | 6 |
| Mou et al., 2018 | Y | Y | | Y | | N | | N | N | N | | N | | N | | N | 1 |
| Nicastro et al., 2016 | Y | Y | | Y | | N | | N | N | Y | | Y | | Y | | Y | 5 |
| Nisenbaum et al., 2010 | Y | Y | | Y | | N | | Y | N | Y | | Y | | Y | | Y | 6 |
| Nurnberg et al., 1986 | Y | Y | | Y | | N | | Y | N | Y | | Y | | Y | | Y | 6 |
| Nurnberg et al., 1987 | Y | Y | | Y | | N | | Y | N | Y | | Y | | Y | | Y | 6 |
| Nurnberg et al., 1991 | Y | Y | | N | | N | | Y | N | Y | | Y | | Y | | Y | 5 |
| Ohshima, 2001 | Y | Y | | Y | | N | | Y | N | Y | | Y | | Y | | Y | 6 |
| Oldham et al., 1996 | Y | Y | | Y | | N | | N | N | Y | | N | | Y | | Y | 4 |
| Perez, et al., 2014 | Y | Y | | Y | | N | | Y | N | Y | | Y | | Y | | Y | 6 |
| Perroud et al., 2013 | Y | Y | | Y | | N | | Y | N | Y | | Y | | Y | | Y | 6 |
| Pinto et al., 1996 | Y | Y | | Y | | N | | Y | N | Y | | Y | | Y | | Y | 6 |
| Powers et al., 2013 | Y | Y | | N | | Y | | Y | N | Y | | Y | | Y | | Y | 6 |
| Price et al., 2019 | Y | Y | | N | | N | | Y | N | Y | | Y | | Y | | Y | 5 |
| Rebok et al., 2015 | Y | Y | | Y | | N | | Y | Y | N | | N | | N | | Y | 4 |
| Richman & Sokolove, 1992 | Y | Y | | Y | | N | | Y | Y | Y | | Y | | Y | | Y | 7 |
| Rippetoe et al., 1986 | Y | Y | | Y | | N | | Y | N | Y | | N | | Y | | Y | 5 |
| Rogers et al., 1995 | Y | Y | | Y | | N | | Y | N | Y | | Y | | Y | | Y | 6 |
| Sanislow et al., 2000 | Y | Y | | Y | | N | | Y | N | Y | | Y | | Y | | Y | 6 |
| Scheel et al., 2013 | Y | Y | | Y | | N | | N | N | Y | | N | | Y | | Y | 4 |
| Silk et al., 1995 | Y | Y | | Y | | N | | Y | N | Y | | N | | N | | Y | 4 |
| Skinstad et al., 1999 | Y | Y | | Y | | N | | Y | N | Y | | Y | | Y | | Y | 6 |
| Soloff et al., 2002 | Y | Y | | Y | | N | | Y | N | Y | | Y | | Y | | N | 5 |
| Soloff et al., 2000 | Y | Y | | Y | | N | | Y | N | Y | | Y | | Y | | N | 5 |
| Southward & Cheavens, 2018 | Y | Y | | N | | N | | Y | N | Y | | Y | | Y | | Y | 5 |
| Speranza et al., 2012 | Y | Y | | Y | | N | | Y | N | Y | | Y | | Y | | Y | 6 |
| Stanley et al., 2001 | Y | Y | | Y | | N | | Y | N | Y | | Y | | Y | | Y | 6 |
| Stepp et al., 2009 | Y | Y | | Y | | N | | Y | N | Y | | Y | | Y | | Y | 6 |
| Stiglmayr et al., 2005 | Y | Y | | Y | | N | | Y | N | Y | | Y | | N | | Y | 5 |
| Taylor & Reeves, 2007 | Y | Y | | N | | N | | Y | N | Y | | Y | | Y | | Y | 5 |
| Taylor & Goritsas, 1994 | Y | Y | | N | | N | | Y | N | Y | | Y | | Y | | Y | 5 |
| Thome et al., 2016 | Y | Y | | Y | | N | | Y | N | Y | | Y | | Y | | Y | 6 |
| Trull & Widiger, 1991 | Y | Y | | N | | N | | Y | N | Y | | Y | | N | | Y | 4 |
| Verardi et al., 2008 | Y | Y | | Y | | N | | N | N | Y | | Y | | Y | | Y | 5 |
| Vardy et al., 2019 | Y | Y | | Y | | N | | Y | N | Y | | Y | | Y | | Y | 6 |
| Verkes et al., 1998 | Y | Y | | Y | | N | | Y | Y | Y | | Y | | Y | | Y | 7 |
| Villeneuve & Lemelin, 2005 | Y | Y | | Y | | N | | Y | Y | Y | | Y | | Y | | Y | 7 |
| Wedig et al., 2013 | Y | Y | | Y | | N | | Y | Y | Y | | Y | | Y | | Y | 7 |
| Westen et al., 1992 | Y | Y | | Y | | N | | Y | N | Y | | Y | | Y | | Y | 6 |
| Yen et al., 2009 | Y | Y | | Y | | N | | Y | N | Y | | Y | | Y | | Y | 6 |
| Zanarini et al., 1998 | Y | Y | | Y | | N | | Y | Y | Y | | Y | | Y | | Y | 7 |
| Zanarini et al., 2016 | Y | Y | | Y | | N | | Y | N | Y | | Y | | Y | | Y | 6 |
| Zanarini et al., 2007 | Y | Y | | Y | | N | | Y | Y | Y | | Y | | Y | | Y | 7 |
| **Authors (year)** | **Screening questions** | | | | **1.1 Are the sources of qualitative data relevant to address the research question?** | | | | **1.2 Is the process for analysing qualitative data relevant to address the research question?** | | | | **1.3 Is appropriate consideration given to how findings relate to the context e.g. the setting in which data were collected?** | | **1.4 Is appropriate consideration given to how findings relate to researchers' influence e.g. through their interactions with participants?** | | **Quality of study (range 0-6)** |
|  | **Are there clear research questions/ objectives?** | | **Do the collected data address the research question?** | | **Is the selection of participants clear?** | | **Are reasons why potential participants chose not to participate explained?** | | **Is the method of data collection clear and the form of the data is clear?** | | **Does the data analysis address the question?** | |  | |  | |  |
| Ntshingila et al., 2016 | Y | | Y | | Y | | N | | Y | | Y | | Y | | N | | 4 |
| Sagan, 2017 | Y | | Y | | Y | | N | | Y | | Y | | N | | N | | 3 |
| Vardy et al., 2019 | Y | | Y | | Y | | N | | Y | | Y | | N | | N | | 3 |
